# Genome-wide association study reveals genetic loci and candidate genes for meat quality traits in a four-way crossbred pig population

**DOI:** 10.3389/fgene.2023.1001352

**Published:** 2023-02-06

**Authors:** Huiyu Wang, Xiaoyi Wang, Mingli Li, Hao Sun, Qiang Chen, Dawei Yan, Xinxing Dong, Yuchun Pan, Shaoxiong Lu

**Affiliations:** ^1^ Faculty of Animal Science and Technology, Yunnan Agricultural University, Kunming, Yunnan, China; ^2^ Faculty of Animal Science, Xichang University, Xichang, Sichuan, China; ^3^ Faculty of Agriculture and Biology, Shanghai Jiao Tong University, Shanghai, China; ^4^ Faculty of Animal Science, Zhejiang University, Hangzhou, Zhejiang, China

**Keywords:** GWAS, crossbred pigs, meat quality, SLAF-seq, candidate genes

## Abstract

Meat quality traits (MQTs) have gained more attention from breeders due to their increasing economic value in the commercial pig industry. In this genome-wide association study (GWAS), 223 four-way intercross pigs were genotyped using the specific-locus amplified fragment sequencing (SLAF-seq) and phenotyped for PH at 45 min *post mortem* (PH45), meat color score (MC), marbling score (MA), water loss rate (WL), drip loss (DL) in the longissimus muscle, and cooking loss (CL) in the psoas major muscle. A total of 227, 921 filtered single nucleotide polymorphisms (SNPs) evenly distributed across the entire genome were detected to perform GWAS. A total of 64 SNPs were identified for six meat quality traits using the mixed linear model (MLM), of which 24 SNPs were located in previously reported QTL regions. The phenotypic variation explained (PVE) by the significant SNPs was from 2.43% to 16.32%. The genomic heritability estimates based on SNP for six meat-quality traits were low to moderate (0.07–0.47) being the lowest for CL and the highest for DL. A total of 30 genes located within 10 kb upstream or downstream of these significant SNPs were found. Furthermore, several candidate genes for MQTs were detected, including pH45 (GRM8), MC (ANKRD6), MA (MACROD2 and ABCG1), WL (TMEM50A), CL (PIP4K2A) and DL (CDYL2, CHL1, ABCA4, ZAG and SLC1A2). This study provided substantial new evidence for several candidate genes to participate in different pork quality traits. The identification of these SNPs and candidate genes provided a basis for molecular marker-assisted breeding and improvement of pork quality traits.

## Introduction

Pork quality is a comprehensive indicator, including meat color, pH, marbling, water-holding capacity, intramuscular fat (IMF), tenderness, etc. (Noidad et al., 2019), which is an important economic factor in the pig industry and has been one of the main objectives in pig breeding programs (Gallardo et al., 2012; Nonneman et al., 2013). In the past, pig breeders have been focused on growth performance but neglected meat quality, resulting in the decline of pork quality. However, due to the fast rise in living standards, consumers favor higher-quality pork. In modern pig breeding, more attention has been paid to improving meat quality traits (MQTs) (Fan et al., 2010). However, it is difficult to genetically improve meat quality using conventional breeding methods because meat quality is measured after slaughter. Previous studies have shown that a lot of pork qualities show low to medium heritability (Lee et al., 2015; Khanal et al., 2019). In the past few years, researchers have been committed to improving meat quality through advanced molecular breeding methods, such as molecular marker assisted selection (MAS) breeding. Recently, many candidate genes affecting MQTs have been reported, including *RYR1*, *PRKAG3*, *PHKG1*, and *IGF2* (Milan et al., 2000; Yu et al., 2008; Škrlep et al., 2010; Ma et al., 2014). To date, a total of 18,011 quantitative trait loci (QTLs) for meat and carcass traits have been accumulated in the pig QTL database (http://www.animalgenome.org/cgi-bin/QTLdb/index, 25 Apr 2022). Among these QTLs, 805, 765, 136, 30, 91, and 1,092 are found to be associated with PH and meat color, marbling score, water holding capacity, cooking loss, and drip loss, respectively. However, most of these QTLs detected by linkage mapping cover large regions of the genome containing hundreds of genes. Furthermore, only a few genes have been successfully applied to improve the MQTs of pigs at present. Consequently, identifying accurate QTL locations and novel candidate genes remains a major challenge.

Genome-wide association study (GWAS) has been increasingly used to identify genomic regions and markers related to quantitative traits more precisely. In recent years, GWAS based on SNP array for MQTs has identified a large number of QTLs and candidate genes (Lee et al., 2012; Luo et al., 2012; Ma et al., 2013; Fabbri et al., 2020; Park et al., 2021). Gao et al. (2021) used the GeneSeek Porcine SNP50K BeadChip for 582 Duroc × (Landrace × Yorkshire) (DLY) commercial pigs to identify genes related to meat-quality traits: thirty-two SNPs and several candidate genes for meat quality were identified. Liu et al. (2015) genotyped 36 Chinese Erhualian pigs and 610 DLY commercial pigs using the Illumina PorcineSNP60K Beadchip, and obtained 35, 985 and 56, 216 high-quality SNPs to perform GWAS for 20 meat quality traits, respectively. Several QTL regions and relevant candidate genes for meat quality traits were detected. However, the SNP array still has disadvantages, for example, that only a small number of known SNPs can be detected, and that marker distribution is biased. Currently, GWAS based on genome-wide sequencing (WGS) is a powerful method to associate genome-wide SNP with meat quality traits (Ji et al., 2018). Wu et al. (2020) used WGS to genotype 30 purebred Qingyu pigs and obtained 18,436,759 filtered SNPs to perform GWAS for meat pH and color. Several SNPs and candidate genes (*CXXC5*, *RYR3*, *BNIP3*, and *MYCT1*) for meat traits were identified. For *Sus Scrofa* with larger genomes, GWAS based on whole-genome sequencing (WGS) is prohibitively expensive. Considering these limitations, specific-locus amplified fragment sequencing (SLAF-seq), a technology based on high-throughput sequencing was developed, which is a cost-effective method for large-scale genotyping (Sun et al., 2013). SLAF-seq technology has the following four significant advantages: the generation of millions of high-density SNP loci covering the whole genome, the ability to detect new SNP loci in unknown mutations, its applicability to any species whether there is a reference genome or not, and the use of representative libraries to reduce sequencing costs. As a consequence, SLAF-seq-based GWAS was successfully applied to detect SNP loci for important quantitative traits in rabbits (Yang et al., 2020), chickens (Wang et al., 2015; Wang et al., 2019; Li et al., 2021), ducks (Xi et al., 2021), and geese (Melak et al., 2021). SLAF-seq has also been successfully used for genotyping of pigs and detected abundant novel mutation sites (Li et al., 2017; Qin et al., 2020). Furthermore, we also identified some genomic regions and several candidate genes for porcine fatness-related and growth-related traits using GWAS based on SLAF-seq technology in our previous studies (Wang et al., 2022a; Wang et al., 2022b).

To produce more genetic variation, A (Duroc×Saba) × [Yorkshire × (Landrace × Saba)] hybrid segregation population was established. As we know, Duroc, Landrace, and Yorkshire pigs are typical lean-type Western commercial breeds widely distributed all over the world and used for commercial production. The shared disadvantage of Western commercial pigs is poor meat quality. However, Chinese native pigs are quite different from Western commercial pigs in meat quality traits. As an invaluable Chinese genetic resource, the fat-type Saba pigs are widely distributed in Yunnan Province, China (Diao et al., 2019), which exhibit high intramuscular fat (IMF) content and superior pork quality. Taking Chinese pig breeds with high meat quality and Western pig breeds with poor meat quality as parents, the hybrid offspring show great differences in meat quality traits and can produce more genetic variation.

Here, we examined 223 four-way crossbred pigs raised under the same environmental conditions for six meat quality traits, including pH at 45 min *post mortem* (pH45), meat color score (MC), marbling score (MA), water loss rate (WL), cooking loss (CL), and drip loss (DL). Subsequently, GWAS based on SLAF-seq was performed, and identified potential loci influencing these traits. The findings served as the foundation for molecular marker-assisted breeding and the improvement for meat quality traits in pigs.

## Materials and methods

### Ethics statement

All of the animals utilized in this study were handled and used in accordance with the standards established by China’s Ministry of Agriculture and Rural Affairs for the care and use of experimental animals. The entire study was given the nod by the Yunnan Agricultural University’s (YNAU, Kunming, China) ethics committee.

### Animals

A four-way crossbred pig population was established as described previously (Wang et al., 2022a; Wang et al., 2022b). In short, 223 four-way crossbred pigs (115 females and 108 males, DSYLS) investigated were offspring of seven hybrid boars (Duroc × Saba, DS) and 37 hybrid sows (Yorkshire × (Landrace × Saba), YLS) from the pigs and broilers breeding farm in Chuxiong City, Yunnan Province, China (Supplementary Figure S1). These pigs were raised under identical dietary and environmental settings, with automatic water intake and unfettered access to food, which were slaughtered in the same abattoir weighing 105.25 ± 15.75 kg. The ear tissues of 223 pigs were sampled.

### Phenotypes

Six meat quality traits were noted after slaughter, including PH45, MC, MA, WL, DL, and CL. The measured muscle samples were from the left side of the carcass. PH45, MC, MA, WL, and DL were measured on the longissimus muscle between the 10th rib and the first lumbar vertebra, and CL was measured on the psoas major muscle. PH45 values were measured at 45 min after slaughter using an automatic pH-STAR. MC (ranging from 1 to 6, 1 presents pale color and 6 presents dark color), and MA (ranging from 1 to 6, 1 presents lack and 6 presents overabundance) were subjectively evaluated according to National Pork Producer Council (NPPC) guidelines. The WL was determined using the filter paper press method as described by Farouk and Wieliczko (2003) with some modifications. Samples were weighed before (Wb) and after (Wa) being subjected to a 35 kg force for 5 min using a pressure instrument (YYW-2, Nanjing Soil Instrument Co., Ltd. Nanjing, China). DL after 24 h storage was measured using a bag method (Honikel, 1987). DL samples were weighed before (Db) and after (Da) being hanged at 4°C for 24 h. Finally, about 20 g cube-like raw meat samples from the psoas major muscle were used to measure CL. The raw was weighed (Cb) and steamed for 30 min. Cooked samples were cooled down to room temperature and re-weighed (Ca). WL, DL, and CL were calculated using the following formula:

$$WL\;\left(\%\right)=\left[\left(Wb-Wa\right)\;/\;Wb\right]\times 100\%$$

$$DL\;\left(\%\right)=\left[\left(Db-Da\right)/\;Db\right]\times 100\%$$

$$CL\;\left(\%\right)=\left[\left(Cb-Ca\right)\;/\;Cb\right]\times 100\%$$

Three measurements of PH45, WL, CL, and DL were taken for each sample. Further analyses were conducted using the averages.

The SAS (SAS Institute, Inc., Cary, NC) MEANS procedure was used to create descriptive statistics for meat quality traits under investigation. Using the R package “ggpubr”, the sample distribution was represented as a frequency distribution histogram. The R function “PerformanceAnalytics” carried out the phenotypic correlation analysis. The genetic correlations and genome heritability for six meat quality traits were estimated using the GCTA software (Yang et al., 2011).

### SLAF library construction and sequencing

SLAF library construction and sequencing were performed as described previously (Wang et al., 2022a; Wang et al., 2022b). In short, using the phenol-chloroform extraction procedure, genomic DNA was isolated from ear tissue samples. Concentration and purity were then determined using the NanodropTM 2000 spectrophotometer (Thermo Scientific, Waltham, MA, USA) and electrophoresis. An electronic digestion prediction experiment used the pig genome (Sscrofa 11.1_102, ftp:/ftp.ensembl.org/pub/release-102/) as the reference genome. *RsaI* and *HaeIII* restriction enzyme combinations were selected to digest eligible genomic DNA according to the selection principle of the enzyme digestion scheme (Sun et al., 2013). The enzyme digested fragment (SLAF tag) was treated by adding single-nucleotide A to the 3′end, and fragments were then ligated to the dual index (Kozich et al., 2013) sequencing adaptors, Adaptor-ligated fragments were then amplified by PCR, purified, pooled, and screened to construct the SLAF library. Meanwhile, to test the validity of the experimental procedure, we also subjected the control genome (*Oryza sativa spp. japonica*; 374.30 Mb; http://rapdb. dna.affrc.go.jp/) to the identical sequencing procedure. Briefly, SLAF library construction and sequencing for each individual was carried out as previously described (Sun et al., 2013) with a few minor modifications: target DNA fragments of sizes from 314 to 344 base pair (bp) were selected as SLAF tags and used for paired-end sequencing on an Illumina HiSeq 2,500 platform (Illumina, Inc., San Diego, CA, USA) at Beijing Biomarker Technologies Corporation in Beijing, China.

Dual-Index software was used to examine the raw SLAF-seq data in order to acquire the raw sequencing reads for each sample (Kozich et al., 2013). After removing the adapter reads, the guanine-cytosine (GC) content and Q30 (Q = −10 × log_10_ *p*) were measured to assess the sequencing accuracy. And then, raw paired-end reads were aligned to the pig reference genome (Sscrofa 11.1_102) using BWA software (Li and Durbin, 2009). Polymorphic SLAFs exhibited sequence polymorphisms between distinct samples.

### Identification of SNPs

SNP throughout the entire genome were generated as described previously (Wang et al., 2022a; Wang et al., 2022b). In short, SNP loci were found based on information from polymorphic SLAF tags using predominantly GATK (McKenna et al., 2010). Based on clean reads mapped to the reference genome, local realignments and base recalibration were conducted, and SNPs were detected using GATK software (McKenna et al., 2010). The SAMtools software (Li et al., 2009) was used to detect SNPs in addition to GATK to guarantee the accuracy of the SNPs detected. As the trustworthy set of SNPs to be subjected to the following analysis, we chose the intersection of SNPs found by both GATK and SAMtools. PLINK two software (Purcell et al., 2007) was utilized to filter SNPs according to minor allele frequency (MAF: 0.05) and integrity (int: 0.8). Ultimately, highly consistent population SNPs were detected for GWAS.

### Genome-wide association study (GWAS)

A GWAS was carried out to identify the underlying SNP loci or genes linked to meat quality traits in four-way crossbred pigs. Based on the filtered SNPs (227,921 SNPs) and six meat quality phenotypic data, an association analysis was carried out. We used mixed linear model (MLM) of GEMMA software (Zhou and Stephens, 2012) to detect the SNPs associated with meat quality traits. The MLM formula of GEMMA software was as follows:

y=Wα+xβ+Zμ+ε

Where y was an n×1 vector of phenotype in the four-way crossbred pig population; x was an n×1 vector of marker genotypes, W was the matrix of population structure calculated by the ADMIXTURE software (Alexander et al., 2009), and Z was the matrix of the kinship relationship calculated using GCTA software (Yang et al., 2011). α was the vector of fixed effects; β were the marker effects; μ was random effects and ε was the vector of residuals. Finally, for each variant site, an association result could be attained. Bonferroni correction (BC) approach (Zhou and Stephens, 2012) was used for multiple tests in the study. Markers with adjusted −log_10_ (*p*) > 5 (control threshold) were regarded to be significant SNPs for meat quality traits (Wang et al., 2022a; Wang et al., 2022b). The threshold *p*-value for genome-wide 1% and 10% significance were 4.39 × 10^−8^ (0.01/227,921) and 4.39 × 10^−7^ (0.1/227,921), respectively, according to the number of filtered SNPs (n = 227,921). A marker was deemed to be significantly related to the target trait if it passed the threshold score or above the threshold −log_10_ *p* given the complexity of the target traits. Finally, the manhattan and Quantile-quantile (Q-Q) plots of GWAS were drawn using the R package “qqman” (Turner, 2014).

### Identification, annotation and functional enrichment analysis of candidate genes

Based on the reference (Xie et al., 2017; Xie et al., 2018), the genes in 10 kb upstream or downstream of significant associated SNPs were considered trait-associated potential candidate genes. Using the Ensembl Sscrofa11.1 database (www.ensembl.org), the relevant information of genes within 10 kb upstream or downstream of each significant SNP was obtained. Using Gene Ontology Consortium (http://geneontology.org), GO annotation results of candidate genes were then obtained. GO and KEGG enrichment analyses were performed based on genes located 10 kb upstream and downstream of significant SNPs using the database for annotation, visualization, and integrated discovery (DAVID v6.8, https://david.ncifcrf.gov/). GO terms and KEGG pathways with the threshold *p*-value ≤ 0.05 were regarded to be significantly enriched.

### Haplotype block analysis

Haplotype block analysis was performed with LDBlockShow software (Dong et al., 2021). LD (*r* ^2^) value between SNP pairs>0.7 was defined as a LD block.

## Results

### Phenotype description and genomic heritability for meat quality traits

The statistical data on the six meat quality traits are shown in Table 1. The mean values for PH45, MC, MA, WL, CL, and DL were 6.16%, 3.26%, 2.91%, 15.95%, 39.30%, and 2.27%, respectively. The coefficient of variation (CV) for the six meat quality traits were 4.96, 14.77 19.22, 21.02, 8.12, and 53.40, respectively. The results, therefore, indicated that four-way crossbred pig populations in meat quality traits, especially DL had extraordinary genetic variation. The genomic heritability estimates based on SNP for six meat-quality traits ranged from 0.07 (CL) to 0.47 (DL). The trait distributions are shown in Supplementary Figure S2.

**TABLE 1:** Phenotype and heritability statistics for six meat quality traits in crossbred pigs.

Trait^a^	N^b^	Min^c^	Max^d^	Mean	SD^e^	CV^f^	h^2^ (SE)^g^
PH45	223	5.12	7.04	6.16	0.31	4.96	0.34 ± 0.15
MC	223	1.50	4.50	3.26	0.48	14.77	0.20 ± 0.14
MA	223	2.00	5.00	2.91	0.56	19.22	0.23 ± 0.13
WL (%)	221	6.80	25.43	15.95	3.35	21.02	0.19 ± 0.12
CL (%)	223	26.73	46.69	39.30	3.19	8.12	0.07 ± 0.12
DL (%)	213	0.14	11.30	2.27	1.21	53.40	0.47 ± 0.18

^a^ CL, cooking loss; DL, Drip loss; MA, marbling score; MC, meat color score; PH45, PH at 45min *post mortem*; WL, water loss rate.

^b^ Number of samples.

^c^ Minimum.

^d^ Maximum.

^e^Standard deviation.

^f^ Coefficient of variation.

^g^ Heritability (standard error).

### Correlation among meat quality traits

The phenotypic correlation coefficients for PH45, MC, MA, WL, CL, and DL are showed in Table 2. The results showed that WL had the strongest positive correlation with CL (*r* = 0.38, *p* < 0.001). WL had the strongest negatively correlated with PH45 (*r* = −0.22, *p* < 0.001). The six meat quality traits showed low to medium phenotypic correlation (0.01<|*r* |<0.38), indicating that there was no strong phenotypic correlation between the six meat quality traits. The genetic correlations among six meat quality traits are shown in Table 3.

**TABLE 2:** Phenotypic correlations for six meat quality traits in crossbred pigs.

Trait^a^	PH45	MC	MA	WL	CL
MC	0.11				
MA	0.14*	0.18**			
WL	−0.22***	0.06	0.09		
CL	−0.19**	−0.05	0.07	0.38***	
DL	−0.11	0.02	0.13*	−0.01	0.14*

^a^ CL, cooking loss; DL, Drip loss; MA, marbling score; MC, meat color score; PH45, PH, at 45 min *post mortem*; WL, water loss rate. Negative values represented negative correlation, and positive values represented positive correlation. * significant at *p* < 0.05, ** significant at *p* < 0.01, *** significant at *p* < 0.001.

**TABLE 3:** Genetic correlations for six meat quality traits in crossbred pigs.

Trait^a^	PH45 T28	MC T29	MA T30	WL T58	CL T32
MC T29	0.49 (0.34)				
MA T30	0.53 (0.26)	**1.00 (0.42)**			
WL T58	−0.48 (0.12)	0.21 (0.23)	0.20 (0.20)		
CL T32	−0.50 (0.17)	−0.09 (0.29)	0.09 (0.27)	**1.00 (0.20)**	
DL T34	**−1.00 (1.66)**	−0.07 (0.49)	0.78 (0.43)	−0.07 (0.33)	0.33 (0.29)

^a^ CL, cooking loss; DL, Drip loss; MC, meat color score; MA, marbling score; PH45, PH, at 45 min *post mortem*; WL, water loss rate. Negative values represented negative correlation, and positive values represented positive correlation. The numbers in brackets were standard errors. The extreme values of genetic correlations for meat quality traits were in bold.

### Identification of SLAFs and SNPs

A total of 223 individuals were genotyped and descriptive statistics of the sequence data were presented in our previous study (Wang et al., 2022a; Wang et al., 2022b). In short, a total of 1,190.92 million paired-end reads were obtained. The average value of Q30 and GC content were 90.74% and 44.83%, respectively (Supplementary Table S1), demonstrating that our sequencing results were reliable. Furthermore, a total of 1,552,377 SLAF tags were identified, with 331,608 average SLAFs for accessions. The average sequencing depth of accessions was 11.94 fold (Supplementary Table S2), which guaranteed the accuracy of subsequent analysis. In addition, *Oryza sativa indica* was used as a control during sequencing. The results showed that the enzyme digestion normally efficiency and paired-end comparison efficiency of control data were 90.77% and 95.4%, respectively, indicating that the construction of SLAF libraries was normal.

After genomic mapping and SNP calling, a total of 16,997 polymorphic SLAFs were detected across the accessions. Furthermore, 10,784,484 SNPs in all were identified for all individuals. Based on the selection criteria (integrity>0.8; MAF>0.05), a series of quality control filtering of SNPs was carried out to identify 227,921 SNPs used in the subsequent study. Supplementary Figure S3 displayed the density distribution of the filtered and total SNPs across the entire pig genome. SNPs were found in almost all of the non-overlapping 1 Mb regions of the genome. The density distribution of total SNPs and filtered SNPs were calculated on each *Sus Scrofa* autosome and are shown in Table 4. The filtered SNP density across the 18 *Sus Scrofa* chromosomes was one SNP every 10.28 kb on average, demonstrating the data was reliable.

**TABLE 4:** SNPs distribution on each *Sus Scrofa* chromosome.

Chromosome	Chromosome length (Mb)	Total SNPs	Filtered SNPs	Density of filtered SNPs (kb)
1	274.33	962,754	20,243	13.55
2	151.94	660,827	13,273	11.45
3	132.85	664,042	13,050	10.18
4	130.91	574,489	12,588	10.40
5	104.53	482,531	9709	10.77
6	170.84	824,442	16,685	10.24
7	121.84	591,418	12,173	10.01
8	138.97	558,536	12,296	11.30
9	139.51	634,613	13,944	10.01
10	69.36	418,236	9,101	7.62
11	79.17	386,093	8,224	9.63
12	61.60	390,815	7,156	8.61
13	208.33	729,800	15,140	13.76
14	141.76	662,647	13,709	10.34
15	140.41	546,445	12,131	11.57
16	79.94	363,968	8,639	9.25
17	63.49	364,104	7,783	8.16
18	55.98	302,386	6,760	8.28
Average	125.88	562,119	11,811	**10.28**

SNP, density was presented as the average physical distance between two adjacent SNP loci.

The extreme values of genetic correlations for meat quality traits were in bold.

### Genome-wide association study and identification of candidate genes

**FIGURE 1:**
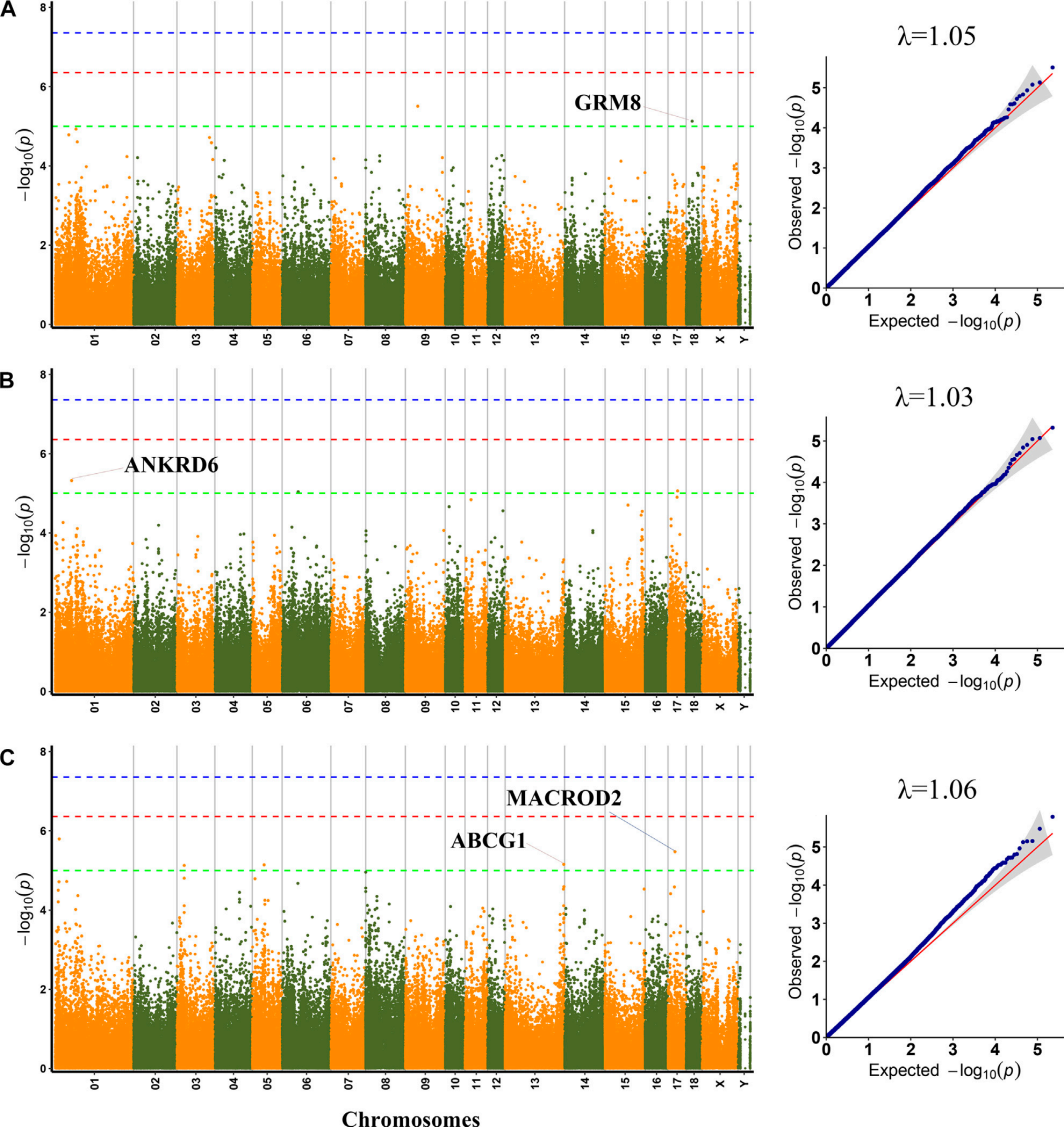
Manhattan plots and QQ plots for pH45, MC and MA using MLM. **(A)** pH45 **(B)** MC **(C)** MA. Negative log_10_ *p*-value of the filtered high-quality SNPs were plotted against their genomic positions; The dashed lines of green, orange and blue correspond to the Bonferroni-corrected thresholds of *p* = 1.00 × 10^−5^ (−log_10_ *p* = 5), *p* = 4.39 × 10^−7^ (−log_10_ *p* = 6.36), and *p* = 4.39 × 10^−8^ (−log_10_ *p* = 7.36), respectively; λ, Genomic inflation factor.

**FIGURE 2:**
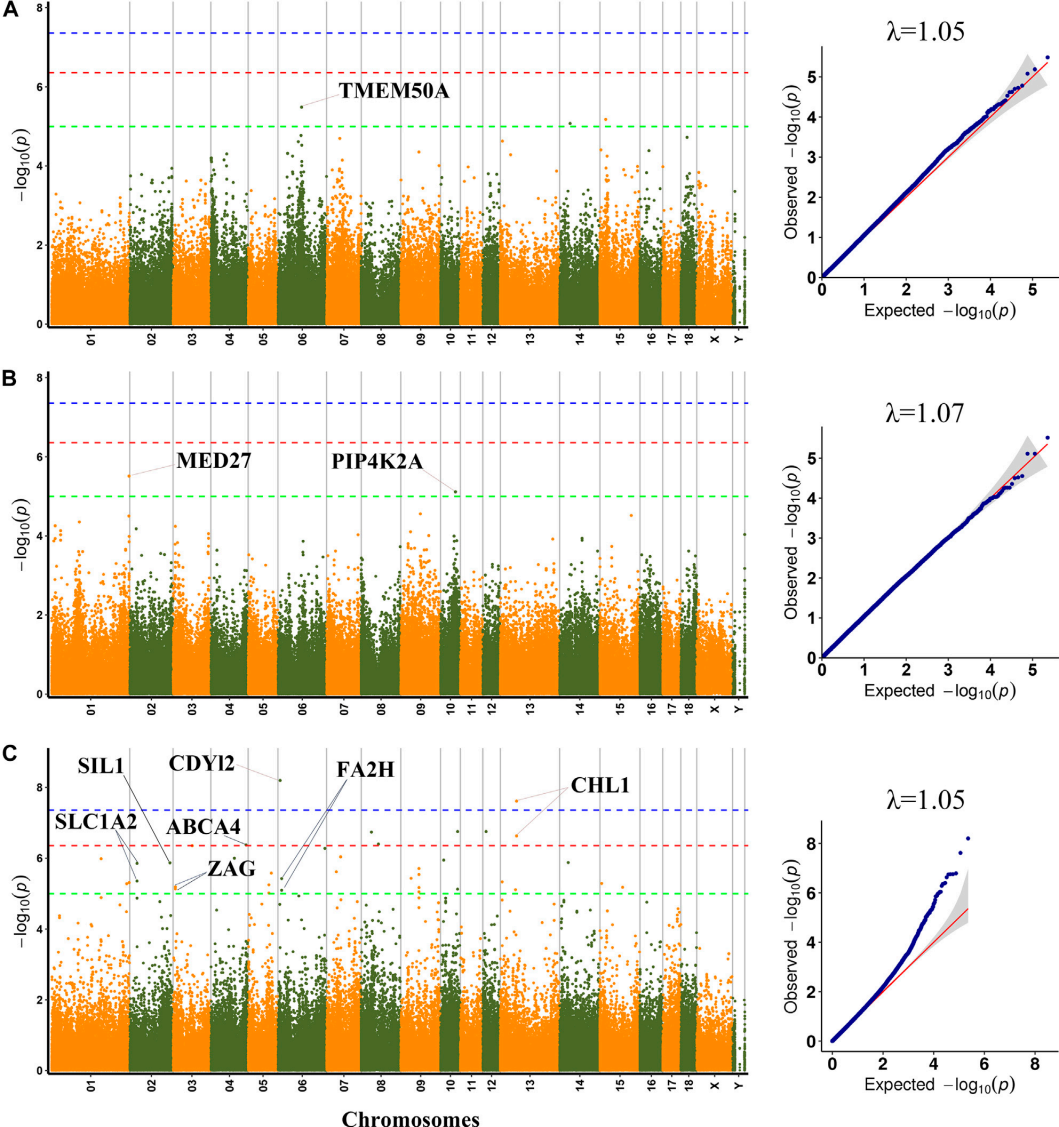
Manhattan plots and QQ plots for WL, CL and DL using MLM. **(A)** WL **(B)** CL **(C)** DL. Negative log_10_ *p*-value of the filtered high-quality SNPs were plotted against their genomic positions; The dashed lines of green, orange and blue correspond to the Bonferroni-corrected thresholds of *p* = 1.00 × 10^−5^ (−log_10_ *p* = 5), *p* = 4.39 × 10^−7^ (−log_10_ *p* = 6.36) and *p* = 4.39 × 10^−8^ (−log_10_ *p* = 7.36), respectively; λ, Genomic inflation factor.

To lessen the impact of population structure and boost the accuracy of GWAS results, the MLM was used to perform GWAS for six meat quality traits. GWAS could be impacted by population stratification, hence quantile-quantile (Q−Q) plots of six meat quality traits were drawn. The Q−Q plot of each trait was shown following the Manhattan plot of the corresponding traits (Figures 1, 2). A total of 64 SNPs were identified as significant (*p* < 1.0 × 10^−5^) for the traits studied using MLM (Supplementary Table S3). The genomic inflation factor (*λ*) at each trait ranged from 1.03 to 1.07.

Among the detected SNPs, three, three, five, three, three, and forty-seven SNPs were significantly associated with PH45, MC, MA, WL, CL and DL, respectively. For pH45, SNPs were distributed in SSC9 (SSC for *Sus scrofa* chromosome), and SSC18. For MC, SNPs were distributed in SSC1, SSC6 and SSC17. For MA, SNPs were distributed in SSC1, SSC3, SSC5, and SSC13. For WL, SNPs were distributed in SSC6, SSC14, and SSC15. For CL, SNPs were distributed in SSC1 and SSC10. For DL, SNPs were distributed in 14 chromosomes except for SSC11, SSC16, SSC17, and SSC18. The phenotypic variation explained (PVE) by the significant SNPs was from 2.43% to 16.32%. Furthermore, 30 genes were thought to be potential candidate genes that were located within 10 kb up- or down-stream of these significant SNPs (Supplementary Table S3).

### pH45

GWAS results showed that three SNP loci identified were significantly related to PH45. Among them, the SNP (SSC9:43364767) was not located in any genes. The significant SNP (rs321002713) on SSC18 explained 11.32% phenotypic variance, which was located within *GRM8*, a protein-coding gene.

### MC and MA

A total of three SNPs were significantly associated with MC. The two significant SNPs, rs327814455 on SSC1 and rs690751971 on SSC6, were located within *ANKRD6* and *ENSSSCG00000032113*, respectively. Among, the rs327814455 explained 10.75% phenotypic variance.

For MA, the most significant SNP (rs696643958) on SSC1 was located within *ENSSSCG00000004081*. The significant SNP (rs341748571) on SSC17 explained 10.47% phenotypic variance, which was located in the *MACROD2* gene. The SNP rs325690789 on SSC5 was located within *FGD4*, and rs342013877 on SSC13 was located 5 kb upstream of the *ABCG1* gene.

### WL and CL

A total of three SNPs (rs1113389876, SSC14:36676133 and SSC15: 19876509) were significantly associated with WL. The most significant SNP (rs1113389876) on SSC6 was located within the *TMEM50A* gene and 7.9 kb upstream of the *RHCE* gene. The significant SNP (rs693644154) on SSC15 was located 2.7 kb upstream of the *RRM2* gene.

**FIGURE 3:**
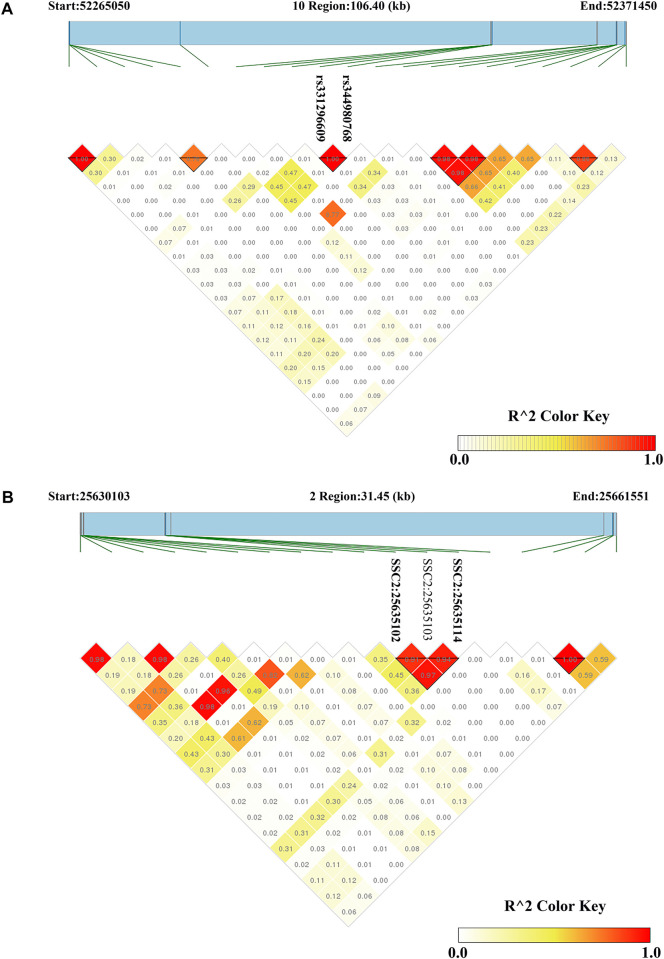
Haploview plots of linkage disequilibrium (r2) between SNPs on pig chromosome. **(A)** A region on SSC10 (106.40 kb) contained a haploview block with two significant SNPs related to CL **(B)** A region on SSC2 (31.45 kb) contained a haploview block with two significant SNPs related to DL. Values in the diamond are *r* ^2^ values between SNPs. The darker the color, the stronger the correlation between two SNPs.

For CL, the most significant SNP (SSC1: 271857436) was located within the *MED27* gene. Furthermore, two nearby significant SNPs (rs331296609 and rs344980768) on SSC10 were located in the *PIP4K2A* gene. These two SNPs were mapped to one haplotype block spanning 16 bp affecting CL on SSC10 (Figure 3A), which each explained 2.43% of the CL phenotypic variance.

### DL

A total of 47 significant SNPs were identified for DL. Among these SNPs, two SNPs (rs321165533 on SSC6 and rs323693055 on SSC13) exceeded the 1% genome-wide significance level. The SNP rs321165533 explained 14.55% phenotypic variance, which was located within the *CDYL2* gene. Eight SNPs (AEMK02000361.1:578806, rs337747094 on SSC3, rs320599347 on SSC4, rs333401534 and rs327130062 on SSC8, SSC10:59940478, rs326956966 on SSC12, and rs703586532 on SSC13) exceeded the 10% genome-wide significance level. Among these significant SNPs, two nearby SNPs on SSC13 (rs323693055 and rs703586532) were located in the *CHL1* gene. The rs703586532 and rs323693055 explained 11.58% and 13.46% phenotypic variance, respectively. The SNP rs320599347 was located within the *ABCA4* gene.

On SSC6, two adjacent significant SNPs (rs326829022 and rs1112488011) were located within *FA2H*. On SSC3, four nearby significant SNPs were located in a region from 7863132 to 7863391 bp (0.26 kb interval), which were located within the *ZAG* gene. Two adjacent significant SNPs (SSC2:25635102 and SSC2:25635114) were located within *SLC1A2*. The two significant SNPs were mapped to one haplotype block spanning 12 bp affecting DL on SSC2 (Figure 3B), which each explained more than 9% of the DL phenotypic variance. Additionally, the rs327708082 on SSC2 explained the highest DL phenotypic variance (16.32%), which was located within the *SIL1* gene.

Furthermore, several significant SNPs explained more than 13.35% phenotypic variance, which were not located any known genes, including rs345860122 on SSC4 (13.48% PVE for DL), rs324617714 and rs325613231 on SSC7 (13.35% PVE for DL).

### Comparison with previously reported QTL in pigs

The Pig Quantitative Trait Locus (QTL) Database (Pig QTLdb, https://www.animalgenome.org/cgi-bin/QTLdb/SS/index, 25 Apr 2022) was searched based on SNP and QTL locations to evaluate if QTLs linked to meat quality traits in this study repeat any previously reported QTLs. A total of 64 SNPs significantly associated with meat quality traits in four-way crossbred pigs were identified using the MLM, of which 24 SNPs were located in previously reported QTL regions that were associated with the meat quality traits of pigs (Supplementary Table S4). Three QTLs, including 9.35-Mb (262.87–272.22Mb) on SSC1, 5.29-Mb (7.60–12.89Mb) on SSC6, and 0.09-Mb region (63.38–63.47Mb) on SSC9 for DL were identified.

### GO annotation and functional enrichment analysis of candidate genes

The result of GO annotation showed that *ENSSSCG00000004081* participated in muscle cell differentiation and actin filament binding. *ABCG1* was involved in negative regulation of lipid storage, response to lipid and phospholipid homeostasis. The GO annotation results of other genes are shown in Supplementary Table S3.

Furthermore, two GO terms (actin binding and photoreceptor outer segment) and one KEGG pathway (ABC transporters) were significantly enriched (*p-*value ≤ 0.05) (Table 5).

**TABLE 5:** Significant GO terms and KEGG pathways associated with meat quality traits in crossbred pigs (*p* ≤ 0.05).

Terms^a^	ID	Count	*p*-value	Genes
KEGG: ABC transporters	ssc02010	2	0.05	*ABCG1*, *ABCA4*
MF: actin binding	GO:0003779	1	0.0005	*ENSSSCG00000004081*
CC: photoreceptor outer segment	GO:0001750	2	0.05	*ABCA4*, *PIP4K2A*

^a^ CC, cell component; KEGG, kyoto encyclopedia of genes and genomes; MF, molecular function.

## Discussion

In this study, we used SLAF-seq technology to obtain 227,921 highly consistent SNPs. Previous studies have proven the advantage of the SLAF-seq method in the GWAS, genetic diversities analysis, and construction of genetic map for animals and plants (Qi et al., 2014; Li et al., 2017; Qin et al., 2020; Yang et al., 2020; Li et al., 2021; Mandozai et al., 2021). SLAF-seq technology can obtain more genomic variation sites than SNP chips, detect novel mutation sites and provide high SNP coverage at a low cost. However, SLAF-seq technology obtains fewer numbers of molecular markers compared with WGS technology. In further study, we used genome re-sequencing technology to attain genome-wide genetic variation, and provided opportunities for understanding more comprehensively and accurately the genetic architecture of pig meat quality traits. Furthermore, these SNPs were used to calculate genetic parameters for six meat quality traits. The genomic heritability estimates based on SNP for six meat-quality traits were low or moderate (0.07–0.47) (Table 1), which was similar to the results of previous studies (Lo et al., 1992; Miar et al., 2014; Gao et al., 2021). The results showed that these meat-quality traits could be genetically improved. There were a high negative genetic correlation (−1.00 ± 1.66) between DL with pH45 and a positive correlation (0.33 ± 0.29) between DL with CL (Table 3), which was similar to the results of the previous studies (Gjerlaug-Enger et al., 2010; Miar et al., 2014). In addition, there were a high positive genetic correlation between MC and MA (1.00 ± 0.42), which was similar to the results of a previous study (Gjerlaug-Enger et al., 2010). There were a high positive genetic correlation (1.00 ± 0.20) between WL and CL, was similar to results of a previous study (Fernández-Barroso et al., 2020). Besides, they had the highest phenotypic correlation (*r* = 0.38; *p* < 0.001).

The standard deviation (SD) of phenotypic values for PH45, MC, and MA were 0.31, 0.48, and 0.56, respectively, which were similar to the results of Gao et al. (2021). Gao et al. found that SD for PH45, MC, and MA were 0.37, 0.55 and 0.61, respectively, in a three-way crossbred commercial pig population. The SD for WL and CL was 3.35 and 3.19, respectively, which were less than the previous studies, including 5.3 for WL in a Korean Native × Landrace F2 cross population (Lee et al., 2012), and 4.17 for CL in a specially designed heterogeneous F6 pig population (Ji et al., 2018). The SD for DL was 1.21, which was more than the previous studies, including 0.33 for DL in a White Duroc × Erhualian F2 population (Ma et al., 2013), and 2.0 and 2.26 for DL in a Korean Native × Landrace F2 cross population (Choi et al., 2011; Lee et al., 2012). Interestingly, the phenotypic variation explained (PVE) of all significant SNPs detected in this study is greater than 2.43%. Among them, the PVE of 26 SNPs was even greater than 10%. The higher PVE of these molecular markers implies that these markers could be used in molecular marker-assisted selection and genome selection in pigs to increase pork quality. Besides, the genomic inflation factor (*λ*) at each trait ranged from 1.03 to 1.07 (Table 1), and none of the Q−Q plots showed any sign of inflation, indicating that the MLM effectively controls the false positive result, and effectively lessen the impact of group stratification on GWAS results, which ensure the reliability of GWAS results.

MAF by the significant SNPs was from 0.05 to 0.49 (Supplementary Table S3). Some significant markers had a low MAF (such as rs327708082, MAF = 0.06). The allele with the lowest frequency had large or very small effects on meat quality traits, depending if these allele showed a positive or negative effect. If the allele with the highest frequency has a positive effect, the selection will not work. In view of these problems, further research was needed to carry out.

### Comparison of pig populations used in this study with those used in other studies

In previous studies, most of the SNPs and candidate genes for important economic traits of pigs identified based on GWAS mainly used F2 generation populations, which were generated by crossbreeding local pig breeds from different countries with Western lean pig breeds (Liu et al., 2014; Zhang et al., 2014; Cho et al., 2015; Guo et al., 2020; Liu et al., 2020) and purebred pigs (Xiong et al., 2015; Ding et al., 2019; Fabbri et al., 2020; Fu et al., 2020). The F2 generation population is characterized by segregation of traits, large phenotypic variation and more genetic diversity, which is suitable for GWAS. Some studies use white Duroc × Erhualian F2 hybrid pig population to conduct GWAS on growth, fat, meat quality, muscle fiber, body size and body weight traits (Ma et al., 2013; Qiao et al., 2015; Guo et al., 2017; Ji et al., 2017; Guo et al., 2020), and obtained a large number of mutation sites and candidate genes related to the research traits. Besides, two studies used Large White × Minzhu F2 generation population to perform GWAS on meat quality and external traits (Luo et al., 2012; Wang et al., 2014), and identified some SNP loci and candidate genes related to meat quality and external traits. A F2 intercross between Landrace and Korean native pigs was used to perform GWAS for meat quality traits (Lee et al., 2012; Cho et al., 2015). In the present study, three typical Western lean-type pig breeds, Landrace, Yorkshire and Duroc, were hybridized with Saba pig, a Chinese local fat-type pig breed, to establish (Duroc×Saba) × [Yorkshire × (Landrace × Saba)] hybrid segregation population, which was used to perform GWAS for six meat quality traits. The four-way hybrid pig population has greater phenotypic variation and more genetic diversity, is a more ideal population for GWAS than the two-way hybrid population and the purebred pig population.

### QTLs identified for meat quality traits

In the present study, 64 SNPs in all were detected using MLM as significant for the meat quality traits studied, of which 24 SNPs were located in previously reported QTL regions for meat quality traits in pigs. Three genomic regions, including 9.35-Mb (262.87–272.22Mb, 3SNPs) on SSC1, 5.29-Mb (7.60–12.89Mb, 3SNPs) on SSC6, and 0.09-Mb region (63.38–63.47Mb, 4SNPs) on SSC9 for DL were located in previously reported QTL regions on SSC1, 6 and 9 for DL (Malek et al., 2001; Thomsen et al., 2004; Liu et al., 2008). Besides, some significant SNPs overlapped with previously reported QTL regions on SSC9 and SSC18 for pH (Harmegnies et al., 2006; Edwards et al., 2008), on SSC6 for MC (Edwards et al., 2008; Li et al., 2010), on SSC1, 5 and 17 for MA (Rohrer et al., 2005; Cho et al., 2015), on SSC6 for water holding capacity (Su et al., 2004). Among SNPs, 40 SNPs had not been included in any previously reported QTLs for meat quality traits (Supplementary Table S4). Two novel QTLs significantly associated with DL, including a 0.08-Mb region (72.91–72.99Mb) on SSC5, a 3.6-Mb region (53.28–56.88Mb) on SSC13 (Supplementary Table S4). In different studies, depending on the specific genetic backgrounds and sample size, different QTLs may be mapped. Moreover, measuring the phenotype of pork quality is a challenge, and different studies may not be measuring exactly the same location of the muscle for meat quality traits. This could contribute to the differences between studies.

Additionally, a 0.36-Mb region (271.86–272.22Mb) on SSC1 was identified as being significantly associated with CL and DL, containing SSC1:271857436 for CL, and rs710333950 and rs326037487 for DL (Supplementary Table S4). A 9.08-Mb region (24.41–34.49 Mb) on SSC17 was identified as being significantly associated with MC and MA, containing rs341748571 for MA, and rs1112200844 for MC (Supplementary Table S4). The findings suggested that certain chromosomal regions might have varying effects on different meat quality traits. Low phenotypic correlation coefficients (*r* = 0.14; *p* < 0.05) and low genetic correlation (0.33 ± 0.29) (Table 3) between CL and DL were founded. Furthermore, low phenotypic correlation coefficients (*r* = 0.18; *p* < 0.01) and High genetic correlation (1 ± 0.42) (Table 3) between MC and MA were founded. As a result, the correlation between the two traits might help to partially account for the pleiotropic effects in the region.

### Candidate genes for six meat quality traits

#### Candidate genes for pH45

Pork pH can affect the quality of meat. Abnormal pork pH will lead to the production of PSE (Pale, Soft, Exudative) or DFD (Dark, Firm, Dry) meat. We identified three significant SNPs as being significantly associated with pH45. Among which, the significant SNP (rs321002713) on SSC18 was located within glutamate metabotropic receptor 8 (*GRM8*). The *GRM8* gene encodes a G protein-coupled metabotropic glutamate receptor involved in glutamatergic neurotransmission in the central nervous system (Nakanishi, 1994; Duvoisin et al., 1995). Group III of the eight different metabotropic glutamate receptors, which are connected to the suppression of the cyclic AMP cascade, includes the GRM8 receptor. (Nakanishi, 1992). A study finds that *GRM8* is a porcine candidate gene related to muscling and a SNP in the *GRM8* gene also displayed a strong association with the loin eye area of pigs (Li et al., 2011). *GRM8* was also associated with the relative area of *longissimus dorsi* muscle fiber type I and was considered a plausible candidate gene for this trait (Guo et al., 2020). Perhaps, the *GRM8* gene expressed in *longissimus dorsi* muscle may be a potential candidate gene for porcine pH traits.

#### Candidate genes for MC

Meat color is a complex trait that depends on the amount of pigment present, the muscle tissue’s structural characteristics, and the pace of muscle acidification (Fan et al., 2008; Mármol-Sánchez et al., 2020). The significant SNP (rs327814455) on SSC1 was located within Ankyrin repeat domain-containing protein 6 (*ANKRD6*). *ANKRD6* belongs to the ankyrins gene family. Ankyrins are a family of structural proteins that include binding sites for cytoskeleton proteins and a variety of integral membranes (Gallagher et al., 1997). Ankyrin interactions allow the cytoskeleton to be attached to the plasma membrane (Rubtsov and Lopina, 2000). Van Deveire et al. (2012) have demonstrated that *ANKRD6* is related to the cross-sectional area of human muscle. Particular muscle phenotypes have been linked in certain studies to genetic variations in the Ankyrin genes. A study shows that SNPs in the bovine Ankyrin 1 (*ANK1*) promoter region have been linked to intramuscular fat levels and tenderness of beef (Horodyska et al., 2015). SNPs in pig *ANK1* show relationships with shear force, pH, water-holding capacity, and intramuscular fat (IMF) (Wimmers et al., 2007). In pig muscle with excessive fat, the Ankyrin repeat and sterile alpha motif domain containing 1B (*ANKS1B*) gene was found to be a significantly upregulated expression (Hamill et al., 2012). Additionally, it has been discovered that the expression of Ankyrin repeat domain 1 (*ANKRD1*) in pig muscle correlates with the ultimate pH (Damon et al., 2013). Consequently, the Ankyrin gene *ANKRD6* should be considered a strong candidate gene for the porcine multiple meat quality traits, containing MC.

#### Candidate genes for MA

The marbling score is closely related to intramuscular fat content (IMF). A low marbling score will affect the pork quality and flavor. The most significant SNP (rs696643958) on SSC1 was located within *ENSSSCG00000004081*. GO annotation result showed that the gene participated in muscle cell differentiation and actin filament binding (Supplementary Table S3). The deposition of fat in muscle is closely related to the growth and development of the muscle (Lai et al., 2004). Thus, the gene may be involved in growth of the muscles and thus affect the fat deposition. On SSC17, one significant SNP (rs341748571) was located within Mono-ADP ribosylhydrolase 2 (*MACROD2*). The *MACROD2* gene encodes the mono-ADP-ribosyltransferase two catalyzing ADP-ribosylation (Feijs et al., 2013). ADP-ribosylation is a post-translational modification participating in a number of biological processes, such as the regulation of transcription, immune cell function, and DNA repair (Kraus and Hottiger, 2013). Some studies find the *MACROD2* gene located at BTA13 which is related to net meat weight in beef cattle (Niu et al., 2021) and may also be affected meat color traits in Nellore cattle (Marin-Garzon et al., 2021). Besides, Ma et al. (2019) find that the *MACROD2* gene may affect porcine backfat thickness traits by affecting fat metabolism. Therefore, the *MACROD2* gene can be considered a candidate gene for the porcine MA.

Another significant SNP (rs342013877) on SSC13 was located 5 kb away from ATP binding cassette subfamily G member 1 (*ABCG1*). In the study, GO annotation results showed that the *ABCG1* gene was involved in negative regulation of lipid storage, response to lipid and phospholipid homeostasis. The *ABCG1* gene has been known to be associated with controlling cellular lipid levels (Kennedy et al., 2005). Adipocyte *ABCG1* can promote lipid accumulation by regulating the lipoprotein lipase (LPL) bioavailability and fat mass growth in a triglyceride (TG)-rich environment (Frisdal et al., 2015). Thus, the *ABCG1* gene also can be considered a strong candidate gene for the pork MA based on its biological functions.

#### Candidate genes for WL

Pork WL is closely related to the water holding capacity of meat, which is affected by the speed and degree of pH decline, protein hydrolysis and even protein oxidation post-mortem (Huff-Lonergan and Lonergan, 2005). The MLM identified the most significant SNPs on SSC6 for WL and the SNP was located in Transmembrane protein 50A (*TMEM50A*). A study shows that the related gene *TMEM217* is associated with meat color (Ma et al., 2013). Besides, in mice, adipocyte metabolism and differentiation are impacted by the related genes *TMEM120A* and *TMEM120B*, which are significantly expressed in fat (Batrakou et al., 2015). Additionally, *TMEM60 and TMEM236* are two other homologous genes related to marbling fat and fat color in cattle, respectively (Lim et al., 2014). Although no studies have shown that *TMEM50A* played a role in meat quality, it might be regarded as a possible candidate gene for WL. The significant SNP on SSC15 was located 2.7 kb upstream of ribonucleotide reductase regulatory subunit M2 (*RRM2*). The result of GO annotation showed that *RRM2* was involved in deoxyribonucleotide biosynthetic process and oxidation-reduction process (Supplementary Table S3). A study finds that inhibitors of *RRM2* can inhibit cell proliferation (Heidel et al., 2007). At present, there was no direct evidence to prove that *RRM2* was related to WL.

#### Candidate genes for CL

The CL can affect the juiciness and appearance of the pork (Aaslyng et al., 2003). The two adjacent SNPs on SSC10 for CL were located within phosphatidylinositol-5-phosphate 4-kinase type 2 alpha (*PIP4K2A*). Previous studies have shown that the two SNPs (ASGA0048292 and ASGA0048295) of *PIP4K2A* were associated with meat quality of pigs (Lee et al., 2014). *PIP4K2A* is related to the fatty acid composition of backfat in three crossbred pigs (Crespo-Piazuelo et al., 2020). The *PIP4K2A* gene controls the body responsiveness to insulin, and mutations in the *PIP4K2A* gene can make the skeletal muscle more sensitive to insulin (Carricaburu et al., 2003). This directly leads to an increase insulin-stimulated glucose transport in muscle (Lamia et al., 2004). Perhaps, *PIP4K2A* might influence meat quality-related traits by affecting glucose transport in muscle. Thus, *PIP4K2A* could be considered a candidate gene for CL.

#### Candidate genes for DL

Drip loss is one of the important indicators to assess pork quality, which is related to ultimate pH, rate of post-mortem pH fall, residual ATP levels, glycolysis rate post-mortem, and activity of several enzymes (Lawrie and Ledward, 2006). The most significant SNP (rs321165533) on SSC6 for DL was located within chromodomain Y-like 2 (*CDYL2*). GO annotation results showed that *CDYL2* was involved in catalytic activity and metabolic processes. A study finds that *CDYL2* is related to porcine teat number (Liu et al., 2022).

Two nearby SNPs (rs703586532 and rs323693055) on SSC13 were located in cell adhesion molecule L1 like (*CHL1*). The study finds that *CHL1* can regulate the cell cycle *via* the p53 pathway and inhibit cell proliferation through the ERK pathway, and was associated with insulin secretion and glucose metabolism (Jiang et al., 2020). Thus, *CHL1* can be considered a strong candidate gene for DL. The SNP rs320599347 on SSC4 was located within ATP binding cassette subfamily A member 4 (*ABCA4*). *ABCA4* is a member of the ABCA subfamily of ATP-binding cassette transporters participating in the transport of phosphatidyle thanolamine (Quazi and Molday, 2013). GO annotation result showed that *ABCA4* participated in phospholipid-translocating ATPase activity, phospholipid translocation, and phospholipid transfer to membrane (Supplementary Table S3). On SSC6, two adjacent significant SNPs were located within fatty acid 2-hydroxylase (*FA2H*), which was participated in fatty acid biosynthetic process, lipid modification, and regulation of cell proliferation (Supplementary Table S3). In 3T3-L1 adipocytes, *FA2H* modulates the diffusional mobility of lipids linked with Raft and lipogenesis (Guo et al., 2010).

Furthermore, four nearby significant SNPs on SSC3 were located in a region of 0.26 kb, which were located within zinc-alpha-2-glycoprotein (*ZAG*), which is a glycoprotein included in the class I family of the major histocompatibility complex (MHC). Several studies show that *ZAG* is related to lipid loss (Bao et al., 2005) and lipid metabolism (Garrido-Sanchez et al., 2012) and also stimulates the expression of adiponectin (Gohda et al., 2003). Besides, two adjacent significant SNPs on SSC2 were located in solute carrier family 1 member 2 (*SLC1A2*). Researchers report that the related genes *SLC15A4* c.658AA genotype has better water-holding capacity (D'Astous-Page et al., 2017). Besides, some previous studies find that genes of the solute carrier family (SLC), such as *SLC25A17* and *SLC9A7* are associated with meat color, drip loss, and intramuscular fat, respectively (Ma et al., 2013), and *SLC37A3* and *SLC24A5* are related to meat color (Iqbal et al., 2015; Gao et al., 2021), and *SLC4A8* and *SLC7A10* are associated with purge loss (Nonneman et al., 2013). In addition, it has been reported that *SLC37A4* and *SLC3A2* are promising candidate genes affecting DL (Ponsuksili et al., 2008; Heidt et al., 2013; Zhao et al., 2019). A large of research suggesting genes of the solute carrier family play important role in regulating DL. Thus, it was inferred that the *SLC1A2* gene could be considered a strong candidate gene for pork DL. Finally, the rs327708082 on SSC2 explained the highest DL phenotypic variance (16.32%), which was located in the SIL1 nucleotide exchange factor (*SIL1*) gene. *SIL1* related to stress protection, and moderately increased *SIL1* also ameliorates cellular fitness under stress conditions (Labisch et al., 2018).

However, more pig populations need to be used to verify these SNP loci and candidate genes, and more pig biological experiments need to be conducted to confirm their functions.

## Conclusion

We conducted a GWAS based on SLAF-seq for six meat-quality traits in 223 four-way crossbred pigs. A total of 64 SNPs distributed on 16 chromosomes were identified using MLM (*p* < 10^–5^), of which 24 SNPs were located in previously reported QTL regions. Three QTLs were identified to be related to DL: 0.08-Mb region on SSC5 (72.91–72.99Mb), 3.6-Mb region on SSC13 (53.28–56.88Mb), and 0.09-Mb region on SSC9 (63.38–63.47Mb). Some novel candidate genes for meat quality traits were identified, including pH45 (*GRM8*), MC (*ANKRD6*), MA (*MACROD2* and *ABCG1*), WL (*TMEM50A*), CL (*PIP4K2A*), and DL (*CDYL2*, *CHL1*, *ABCA4*, *ZAG* and *SLC1A2*). Overall, the study presented substantial new evidence for the involvement of several candidate genes in different pork quality traits. These SNPs and candidate genes identified in the study provided a basis for molecular marker-assisted breeding and improvement for meat quality traits in pigs.

## Data Availability

The datasets presented in this study can be found in online repositories. The names of the repository/repositories and accession number(s) can be found below: https://www.ncbi.nlm.nih.gov/, SRP376933.

## Supplementary material

Supplementary Figure S1 Establishment flow plot of (Duroc×Saba) × [Yorkshire × (Landrace × Saba)] crossbred pig population.

Supplementary Figure S2 Frequency distribution histogram for six meat quality traits. **(A)** PH at 45 min post mortem ( PH45). **(B)** Meat color score (MC). **(C)** Marbling score (MA). **(D)** Water loss rate (WL). **(E)** Cooking loss (CL). **(F)** Drip loss (DL).

Supplementary Figure S3 The density distribution of total SNPs and filtered SNPs on Sus Scrofa chromosomes. **(A)** The number of total SNPs within 1 Mb window size. **(B)** The number of filtered SNPs within 1 Mb window size. The horizontal axis (X-axis) showed the chromosome length (Mb). The color index indicated the number of labels.

## References

[B1] Aaslyng M. D. Bejerholma C. Ertbjergb P. Bertramc H. C. Andersenc H. J. (2003). Cooking loss and juiciness of pork in relation to raw meat quality and cooking procedure. Food Qual. Prefer. 14, 277–288. 10.1016/s0950-3293(02)00086-1

[B2] Alexander D. H. Novembre J. Lange K. (2009). Fast model-based estimation of ancestry in unrelated individuals. Genome Res. 19, 1655–1664. 10.1101/gr.094052.109 19648217 PMC2752134

[B4] Bao Y. Bing C. Hunter L. Jenkins J. R. Wabitsch M. Trayhurn P. (2005). Zinc-alpha2-glycoprotein, a lipid mobilizing factor, is expressed and secreted by human (SGBS) adipocytes. FEBS Lett. 579, 41–47. 10.1016/j.febslet.2004.11.042 15620688

[B5] Batrakou D. G. de Las Heras J. I. Czapiewski R. Mouras R. Schirmer E. C. (2015). TMEM120A and B: Nuclear envelope transmembrane proteins important for adipocyte differentiation. PLoS One 10, e0127712. 10.1371/journal.pone.0127712 26024229 PMC4449205

[B6] Carricaburu V. Lamia K. A. Lo E. Favereaux L. Payrastre B. Cantley L. C. (2003). The phosphatidylinositol (PI)-5-phosphate 4-kinase type II enzyme controls insulin signaling by regulating PI-3,4,5-trisphosphate degradation. Proc. Natl. Acad. Sci. U. S. A. 100, 9867–9872. 10.1073/pnas.1734038100 12897244 PMC187868

[B7] Cho I. C. Yoo C. K. Lee J. B. Jung E. J. Han S. H. Lee S. S. (2015). Genome-wide QTL analysis of meat quality-related traits in a large F2 intercross between Landrace and Korean native pigs. Genet. Sel. Evol. 47, 7. 10.1186/s12711-014-0080-6 25888076 PMC4336478

[B8] Choi B. H. Lee Y. M. Alam M. Lee J. H. Kim T. H. Kim K. S. (2011). Detection of mendelian and parent-of-origin quantitative trait loci for meat quality in a cross between Korean native pig and Landrace. Asian-Aust J. Anim. Sci. 12, 1644–1650. 10.5713/ajas.2011.11166

[B9] Crespo-Piazuelo D. Criado-Mesas L. Revilla M. Castello A. Noguera J. L. Fernandez A. I. (2020). Identification of strong candidate genes for backfat and intramuscular fatty acid composition in three crosses based on the Iberian pig. Sci. Rep. 10, 13962. 10.1038/s41598-020-70894-2 32811870 PMC7435270

[B10] D'Astous-Page J. Gariepy C. Blouin R. Cliche S. Methot S. Sullivan B. (2017). Identification of single nucleotide polymorphisms in carnosine-related genes and effects of genotypes on pork meat quality attributes. Meat Sci. 134, 54–60. 10.1016/j.meatsci.2017.07.019 28759885

[B11] Damon M. Denieul K. Vincent A. Bonhomme N. Wyszynska-Koko J. Lebret B. (2013). Associations between muscle gene expression pattern and technological and sensory meat traits highlight new biomarkers for pork quality assessment. Meat Sci. 95, 744–754. 10.1016/j.meatsci.2013.01.016 23481319

[B12] Diao S. Huang S. Chen Z. Teng J. Ma Y. Yuan X. (2019). Genome-wide signatures of selection detection in three South China indigenous pigs. Genes (Basel) 10, 346. 10.3390/genes10050346 31067806 PMC6563113

[B13] Ding R. Yang M. Quan J. Li S. Zhuang Z. Zhou S. (2019). Single-locus and multi-locus genome-wide association studies for intramuscular fat in Duroc pigs. Front. Genet. 10, 619. 10.3389/fgene.2019.00619 31316554 PMC6609572

[B117] Dong S. S. He W. M. Ji J. J. Zhang C. Guo Y. Yang T. L. (2021). LDBlockShow: A fast and convenient tool for visualizing linkage disequilibrium and haplotype blocks based on variant call format files. Brief Bioinform. 22, bbaa227. 10.1093/bib/bbaa227 33126247

[B14] Duvoisin R. M. Zhang C. Ramonell K. (1995). A novel metabotropic glutamate receptor expressed in the retina and olfactory bulb. J. Neurosci. 15, 3075–3083. 10.1523/JNEUROSCI.15-04-03075.1995 7722646 PMC6577763

[B15] Edwards D. B. Ernst C. W. Raney N. E. Doumit M. E. Hoge M. D. Bates R. O. (2008). Quantitative trait locus mapping in an F2 Duroc x pietrain resource population: II. Carcass and meat quality traits. J. Anim. Sci. 86, 254–266. 10.2527/jas.2006-626 17965326

[B16] Fabbri M. C. Zappaterra M. Davoli R. Zambonelli P. (2020). Genome-wide association study identifies markers associated with carcass and meat quality traits in Italian Large White pigs. Anim. Genet. 51, 950–952. 10.1111/age.13013 33058170

[B17] Fan B. Glenn K. L. Geiger B. Mileham A. Rothschild M. F. (2008). Investigation of QTL regions on chromosome 17 for genes associated with meat color in the pig. J. Anim. Breed. Genet. 125, 240–247. 10.1111/j.1439-0388.2008.00749.x 18717966

[B18] Fan B. Lkhagvadorj S. Cai W. Young J. Smith R. M. Dekkers J. C. (2010). Identification of genetic markers associated with residual feed intake and meat quality traits in the pig. Meat Sci. 84, 645–650. 10.1016/j.meatsci.2009.10.025 20374837

[B118] Farouk M. M. Wieliczko K. J. (2003). Effect of diet and fat content on the functional properties of thawed beef. Meat Sci. 64, 451–458. 10.1016/S0309-1740(02)00214-0 22063127

[B19] Feijs K. L. Forst A. H. Verheugd P. Luscher B. (2013). Macrodomain-containing proteins: Regulating new intracellular functions of mono(ADP-ribosyl)ation. Nat. Rev. Mol. Cell Biol. 14, 443–451. 10.1038/nrm3601 23736681 PMC7097401

[B20] Fernández-Barroso M. Á. Silió L. Rodríguez C. Palma-Granados P. López A. Caraballo C. (2020). Genetic parameter estimation and gene association analyses for meat quality traits in open-air free-range Iberian pigs. J. Anim. Breed. Genet. 137, 581–598. 10.1111/jbg.12498 32761820

[B21] Frisdal E. Le Lay S. Hooton H. Poupel L. Olivier M. Alili R. (2015). Adipocyte ATP-binding cassette G1 promotes triglyceride storage, fat mass growth, and human obesity. Diabetes 64, 840–855. 10.2337/db14-0245 25249572

[B22] Fu L. Jiang Y. Wang C. Mei M. Zhou Z. Jiang Y. (2020). A genome-wide association study on feed efficiency related traits in Landrace pigs. Front. Genet. 11, 692. 10.3389/fgene.2020.00692 32719719 PMC7350416

[B23] Gallagher P. G. Tse W. T. Scarpa A. L. Lux S. E. Forget B. G. (1997). Structure and organization of the human ankyrin-1 gene. Basis for complexity of pre-mRNA processing. J. Biol. Chem. 272, 19220–19228. 10.1074/jbc.272.31.19220 9235914

[B24] Gallardo D. Pena R. N. Quintanilla R. Ramirez O. Almuzara D. Noguera J. L. (2012). Quantitative trait loci analysis of a Duroc commercial population highlights differences in the genetic determination of meat quality traits at two different muscles. Anim. Genet. 43, 800–804. 10.1111/j.1365-2052.2012.02333.x 22497576

[B25] Gao G. X. Gao N. Li S. C. Kuang W. J. Zhu L. Jiang W. (2021). Genome-wide association study of meat quality traits in a three-way crossbred commercial pig population. Front. Genet. 12, 614087. 10.3389/fgene.2021.614087 33815461 PMC8010252

[B26] Garrido-Sanchez L. García-Fuentes E. Fernández-García D. Escote X. Alcaide J. Perez-Martinez P. (2012). Zinc-alpha 2-glycoprotein gene expression in adipose tissue is related with insulin resistance and lipolytic genes in morbidly obese patients. PLoS One 7, e33264. 10.1371/journal.pone.0033264 22442679 PMC3307730

[B27] Gjerlaug-Enger E. Aass L. Odegård J. Vangen O. (2010). Genetic parameters of meat quality traits in two pig breeds measured by rapid methods. Animal 4, 1832–1843. 10.1017/S175173111000114X 22445144

[B28] Gohda T. Makita Y. Shike T. Tanimoto M. Funabiki K. Horikoshi S. (2003). Identification of epistatic interaction involved in obesity using the KK/Ta mouse as a type 2 diabetes model: Is Zn-α2 glycoprotein-1 a candidate gene for obesity? Diabetes 52, 2175–2181. 10.2337/diabetes.52.8.2175 12882938

[B29] Guo L. Zhou D. Pryse K. M. Okunade A. L. Su X. (2010). Fatty acid 2-hydroxylase mediates diffusional mobility of Raft-associated lipids, GLUT4 level, and lipogenesis in 3T3-L1 adipocytes. J. Biol. Chem. 285, 25438–25447. 10.1074/jbc.M110.119933 20519515 PMC2919107

[B30] Guo T. Gao J. Yang B. Yan G. Xiao S. Zhang Z. (2020). A whole genome sequence association study of muscle fiber traits in a White Duroc × Erhualian F2 resource population. Asian-Australas J. Anim. Sci. 33, 704–711. 10.5713/ajas.18.0767 31480184 PMC7206406

[B31] Guo Y. Huang Y. Hou L. Ma J. Chen C. Ai H. (2017). Genome-wide detection of genetic markers associated with growth and fatness in four pig populations using four approaches. Genet. Sel. Evol. 49, 21. 10.1186/s12711-017-0295-4 28196480 PMC5307927

[B32] Hamill R. M. McBryan J. McGee C. Mullen A. M. Sweeney T. Talbot A. (2012). Functional analysis of muscle gene expression profiles associated with tenderness and intramuscular fat content in pork. Meat Sci. 92, 440–450. 10.1016/j.meatsci.2012.05.007 22688437

[B33] Harmegnies N. Davin F. De Smet S. Buys N. Georges M. Coppieters W. (2006). Results of a whole-genome quantitative trait locus scan for growth, carcass composition and meat quality in a porcine four-way cross. Anim. Genet. 37, 543–553. 10.1111/j.1365-2052.2006.01523.x 17121599

[B34] Heidel J. D. Liu J. Y.-C. Yen Y. Zhou B. Heale B. S. Rossi J. J. (2007). Potent siRNA inhibitors of ribonucleotide reductase subunit RRM2 reduce cell proliferation *in vitro* and *in vivo* . Clin. Cancer Res. 13, 2207–2215. 10.1158/1078-0432.CCR-06-2218 17404105

[B35] Heidt H. Cinar M. U. Uddin M. J. Looft C. Jungst H. Tesfaye D. (2013). A genetical genomics approach reveals new candidates and confirms known candidate genes for drip loss in a porcine resource population. Mamm. Genome 24, 416–426. 10.1007/s00335-013-9473-z 24026665

[B36] Honikel K. O. (1987). The water binding of meat. Fleischwirtzchaft 67, 1098–1102.

[B37] Horodyska J. Sweeney T. Ryan M. Hamill R. M. (2015). Novel SNPs in the Ankyrin 1 gene and their association with beef quality traits. Meat Sci. 108, 88–96. 10.1016/j.meatsci.2015.04.019 26051041

[B38] Huff-Lonergan E. Lonergan S. M. (2005). Mechanisms of water-holding capacity of meat: The role of postmortem biochemical and structural changes. Meat Sci. 71, 194–204. 10.1016/j.meatsci.2005.04.022 22064064

[B39] Iqbal A. Kim Y. S. Kang J. M. Lee Y. M. Rai R. Jung J. H. (2015). Genome-wide association study to identify quantitative trait loci for meat and carcass quality traits in Berkshire. Asian-Australas J. Anim. Sci. 28, 1537–1544. 10.5713/ajas.15.0752 26580276 PMC4647092

[B40] Ji J. Zhou L. Guo Y. Huang L. Ma J. (2017). Genome-wide association study identifies 22 new loci for body dimension and body weight traits in a White Duroc × Erhualian F2 intercross population. Asian-Australas J. Anim. Sci. 30, 1066–1073. 10.5713/ajas.16.0679 28111436 PMC5494478

[B41] Ji J. Zhou L. Huang Y. Zheng M. Liu X. Zhang Y. A. (2018). A whole-genome sequence based association study on pork eating quality traits and cooking loss in a specially designed heterogeneous F6 pig population. Meat Sci. 146, 160–167. 10.1016/j.meatsci.2018.08.013 30153624

[B42] Jiang H. Liu Y. Qian Y. Shen Z. He Y. Gao R. (2020). CHL1 promotes insulin secretion and negatively regulates the proliferation of pancreatic β cells. Biochem. Biophys. Res. Commun. 525, 1095–1102. 10.1016/j.bbrc.2020.03.040 32184019

[B43] Kennedy M. A. Barrera G. C. Nakamura K. Baldán Á. Tarr P. Fishbein M. C. (2005). ABCG1 has a critical role in mediating cholesterol efflux to HDL and preventing cellular lipid accumulation. Cell Metab. 1, 121–131. 10.1016/j.cmet.2005.01.002 16054053

[B44] Khanal P. Maltecca C. Schwab C. Gray K. Tiezzi F. (2019). Genetic parameters of meat quality, carcass composition, and growth traits in commercial swine. J. Anim. Sci. 97, 3669–3683. 10.1093/jas/skz247 31350997 PMC6735811

[B45] Kozich J. J. Westcott S. L. Baxter N. T. Highlander S. K. Schloss P. D. (2013). Development of a dual-index sequencing strategy and curation pipeline for analyzing amplicon sequence data on the MiSeq Illumina sequencing platform. Appl. Environ. Microbiol. 79, 5112–5120. 10.1128/AEM.01043-13 23793624 PMC3753973

[B46] Kraus W. L. Hottiger M. O. (2013). PARP-1 and gene regulation: Progress and puzzles. Mol. Asp. Med. 34, 1109–1123. 10.1016/j.mam.2013.01.005 23357755

[B47] Labisch T. Buchkremer S. Phan V. Kollipara L. Gatz C., (2018). Tracking effects of SIL1 increase: Taking a closer look beyond the consequences of elevated expression level. Mol. Neurobiol. 55, 2524–2546. 10.1007/s12035-017-0494-6 28401474

[B48] Lai K. M. Gonzalez M. Poueymirou W. T. Kline W. O. Na E. Zlotchenko E. (2004). Conditional activation of Akt in adult skeletal muscle induces rapid hypertrophy. Mol. Cell Biol. 24, 9295–9304. 10.1128/MCB.24.21.9295-9304.2004 15485899 PMC522257

[B49] Lamia K. A. Peroni O. D. Kim Y. B. Rameh L. E. Kahn B. B. Cantley L. C. (2004). Increased insulin sensitivity and reduced adiposity in phosphatidylinositol 5-phosphate 4-kinase β−/− mice. Mol. Cell Biol. 24, 5080–5087. 10.1128/MCB.24.11.5080-5087.2004 15143198 PMC416424

[B50] Lawrie R. A. Ledward D. (2006). Lawrie’s meat science. Duxford, UK: Woodhead Publishing in Food Science Technology & Nutrition.

[B51] Lee J. H. Song K. D. Lee H. K. Cho K. H. Park H. C. Park K. D. (2015). Genetic parameters of reproductive and meat quality traits in Korean Berkshire pigs. Asian-Australas J. Anim. Sci. 28, 1388–1393. 10.5713/ajas.15.0097 26323395 PMC4554845

[B52] Lee K. T. Lee Y. M. Alam M. Choi B. Park M. Kim K. S. (2012). A whole genome association study on meat quality traits using high density SNP chips in a cross between Korean native pig and Landrace. Asian-Australasian J. Anim. Sci. 25, 1529–1539. 10.5713/ajas.2012.12474 PMC4093033 25049513

[B53] Lee T. Shin D. H. Cho S. Kang H. S. Kim S. H. Lee H. K. (2014). Genome-wide association study of integrated meat quality-related traits of the Duroc pig breed. Asian-Australas J. Anim. Sci. 27, 303–309. 10.5713/ajas.2013.13385 25049955 PMC4093258

[B54] Li F. Liu J. Liu W. Gao J. Lei Q. Han H. (2021). Genome-wide association study of body size traits in Wenshang Barred chickens based on the specific-locus amplified fragment sequencing technology. J. Anim. Sci. 92, e13506. 10.1111/asj.13506 33398896

[B55] Li H. Durbin R. (2009). Fast and accurate short read alignment with Burrows-Wheeler transform. Bioinformatics 25, 1754–1760. 10.1093/bioinformatics/btp324 19451168 PMC2705234

[B56] Li H. Handsaker B. Wysoker A. Fennell T. Ruan J. Homer N. (2009). The sequence alignment/map format and SAMtools. Bioinformatics 25, 2078–2079. 10.1093/bioinformatics/btp352 19505943 PMC2723002

[B57] Li X. Kim S. W. Choi J. S. Lee Y. M. Lee C. K. Choi B. H. (2010). Investigation of porcine FABP3 and LEPR gene polymorphisms and mRNA expression for variation in intramuscular fat content. Mol. Biol. Rep. 37, 3931–3939. 10.1007/s11033-010-0050-1 20300864

[B58] Li X. Kim S. W. Do K. T. Ha Y. K. Lee Y. M. Yoon S. H. (2011). Analyses of porcine public SNPs in coding-gene regions by re-sequencing and phenotypic association studies. Mol. Biol. Rep. 38, 3805–3820. 10.1007/s11033-010-0496-1 21107721

[B59] Li Z. Wei S. Li H. Wu K. Cai Z. Li D. (2017). Genome-wide genetic structure and differentially selected regions among Landrace, Erhualian, and Meishan pigs using specific-locus amplified fragment sequencing. Sci. Rep. 7, 10063. 10.1038/s41598-017-09969-6 28855565 PMC5577042

[B60] Lim D. Kim N. K. Lee S. H. Park H. S. Cho Y. M. Chai H. H. (2014). Characterization of genes for beef marbling based on applying gene coexpression network. Int. J. genomics 2014, 708562. 10.1155/2014/708562 24624372 PMC3929194

[B61] Liu G. S. Kim J. J. Jonas E. Wimmers K. Ponsuksili S. Murani E. (2008). Combined line-cross and half-sib QTL analysis in Duroc-Pietrain population. Mamm. genome 19, 429–438. 10.1007/s00335-008-9132-y 18712441

[B62] Liu Q. Yue J. Niu N. Liu X. Yan H. Zhao F. P. (2020). Genome-wide association analysis identified BMPR1A as a novel candidate gene affecting the number of thoracic vertebrae in a Large White × Minzhu intercross pig population. Anim. (Basel) 10, 2186. 10.3390/ani10112186 PMC7700692 33266466

[B63] Liu X. Wang L. G. Liang J. Yan H. Zhao K. B. Li N. (2014). Genome-wide association study for certain carcass traits and organ weights in a Large White × Minzhu intercross porcine population. J. Integr. Agr. 13, 2721–2730. 10.1016/s2095-3119(14)60787-5

[B64] Liu X. Xiong X. Yang J. Zhou L. Yang B. Ai H. (2015). Genome-wide association analyses for meat quality traits in Chinese Erhualian pigs and a Western Duroc × (Landrace × Yorkshire) commercial population. Genet. Sel. Evol. 47, 44. 10.1186/s12711-015-0120-x 25962760 PMC4427942

[B65] Liu Z. Li H. Zhong Z. Jiang S. (2022). A whole genome sequencing-based genome-wide association study reveals the potential associations of teat number in Qingping pigs. Anim. (Basel) 12, 1057. 10.3390/ani12091057 PMC9100799 35565484

[B66] Lo L. L. McLaren D. G. McKeith F. K. Fernando R. L. Novakofski J. (1992). Genetic analyses of growth, real-time ultrasound, carcass, and pork quality traits in Duroc and Landrace pigs: II. Heritabilities and correlations. J. Anim. Sci. 70, 2387–2396. 10.2527/1992.7082387x 1506302

[B67] Luo W. Cheng D. Chen S. Wang L. Li Y. Ma X. (2012). Genome-wide association analysis of meat quality traits in a porcine Large White × Minzhu intercross population. Int. J. Biol. Sci. 8, 580–595. 10.7150/ijbs.3614 22532790 PMC3334672

[B68] Ma H. Zhang S. Zhang K. Zhan H. Peng X. Xie S. (2019). Identifying selection signatures for backfat thickness in Yorkshire pigs highlights new regions affecting fat metabolism. Genes (Basel). 10, 254. 10.3390/genes10040254 30925743 PMC6523431

[B69] Ma J. Yang J. Zhou L. Ren J. Liu X. Zhang H. (2014). A splice mutation in the PHKG1 gene causes high glycogen content and low meat quality in pig skeletal muscle. PLoS Genet. 10, e1004710. 10.1371/journal.pgen.1004710 25340394 PMC4207639

[B70] Ma J. Yang J. Zhou L. Zhang Z. Ma H. Xie X. (2013). Genome-wide association study of meat quality traits in a White Duroc × Erhualian F2 intercross and Chinese Sutai pigs. PLoS One 8, e64047. 10.1371/journal.pone.0064047 23724019 PMC3665833

[B71] Malek M. Dekkers J. C. Lee H. K. Baas T. J. Prusa K. Huff-Lonergan E. (2001). A molecular genome scan analysis to identify chromosomal regions influencing economic traits in the pig. II. Meat and muscle composition. Mamm. genome 12, 637–645. 10.1007/s003350020019 11471059

[B72] Mandozai A. Moussa A. A. Zhang Q. Qu J. Du Y. Y. Anwari G. (2021). Genome-wide association study of root and shoot related traits in Spring Soybean (Glycine max L.) at seedling stages using SLAF-Seq. Front. Plant Sci. 12, 568995. 10.3389/fpls.2021.568995 34394134 PMC8355526

[B73] Marin-Garzon N. A. Magalhaes A. F. B. Mota L. F. M. Fonseca L. F. S. Chardulo L. A. L. Albuquerque L. G. (2021). Genome-wide association study identified genomic regions and putative candidate genes affecting meat color traits in Nellore cattle. Meat Sci. 171, 108288. 10.1016/j.meatsci.2020.108288 32949820

[B74] Mármol-Sánchez E. Quintanilla R. Jordana J. Amills M. (2020). An association analysis for 14 candidate genes mapping to meat quality quantitative trait loci in a Duroc pig population reveals that the *ATP1A2* genotype is highly associated with muscle electric conductivity. Anim. Genet. 51, 95–100. 10.1111/age.12864 31633210

[B75] McKenna A. Hanna M. Banks E. Sivachenko A. Cibulskis K. Kernytsky A. (2010). The genome analysis toolkit: A MapReduce framework for analyzing next-generation DNA sequencing data. Genome Res. 20, 1297–1303. 10.1101/gr.107524.110 20644199 PMC2928508

[B76] Melak S. Wang Q. Tian Y. Wei W. Zhang L. Elbeltagy A. (2021). Identification and validation of marketing weight-related SNP markers using SLAF sequencing in male Yangzhou Geese. Genes (Basel). 12, 1203. 10.3390/genes12081203 34440377 PMC8393582

[B77] Miar Y. Plastow G. S. Moore S. S. Manafiazar G. Charagu P. Kemp R. A. (2014). Genetic and phenotypic parameters for carcass and meat quality traits in commercial crossbred pigs. J. Anim. Sci. 92, 2869–2884. 10.2527/jas.2014-7685 24778330

[B78] Milan D. Jeon J. T. Looft C. Amarger V. Robic A. Thelander M. (2000). A mutation in PRKAG3 associated with excess glycogen content in pig skeletal muscle. Science 288, 1248–1251. 10.1126/science.288.5469.1248 10818001

[B79] Nakanishi S. (1994). Metabotropic glutamate receptors: Synaptic transmission, modulation, and plasticity. Neuron 13, 1031–1037. 10.1016/0896-6273(94)90043-4 7946343

[B80] Nakanishi S. (1992). Molecular diversity of glutamate receptors and implications for brain function. Science 258, 597–603. 10.1126/science.1329206 1329206

[B81] Niu Q. Zhang T. Xu L. Wang T. Wang Z. Zhu B. (2021). Integration of selection signatures and multi-trait GWAS reveals polygenic genetic architecture of carcass traits in beef cattle. Genomics 113, 3325–3336. 10.1016/j.ygeno.2021.07.025 34314829

[B82] Noidad S. Limsupavanich R. Suwonsichon S. Chaosap C. (2019). Effect of visual marbling levels in pork loins on meat quality and Thai consumer acceptance and purchase intent. Asian-Australas J. Anim. Sci. 32, 1923–1932. 10.5713/ajas.19.0084 31208188 PMC6819675

[B83] Nonneman D. Shackelford S. King D. Wheeler T. Wiedmann R. Snelling W. (2013). Genome-wide association of meat quality traits and tenderness in swine. J. Anim. Sci. 91, 4043–4050. 10.2527/jas.2013-6255 23942702

[B84] Park J. Lee S. M. Park J. Y. Na C. S. (2021). A genome-wide association study (GWAS) for pH value in the meat of Berkshire pigs. J. Anim. Sci. Technol. 63, 25–35. 10.5187/jast.2021.e17 33987581 PMC7882839

[B85] Percie du Sert N. Ahluwalia A. Alam S. Avey M. T. Baker M. Browne W. J. (2020). Reporting animal research: Explanation and elaboration for the ARRIVE guidelines 2.0. PLoS Biol. 18, e3000411. 10.1371/journal.pbio.3000411 32663221 PMC7360025

[B86] Ponsuksili S. Jonas E. Murani E. Phatsara C. Srikanchai T. Walz C. (2008). Trait correlated expression combined with expression QTL analysis reveals biological pathways and candidate genes affecting water holding capacity of muscle. BMC Genom 9, 367. 10.1186/1471-2164-9-367 PMC2529315 18671879

[B87] Purcell S. Neale B. Todd-Brown K. Thomas L. Ferreira M. A. Bender D. (2007). Plink: A tool set for whole-genome association and population-based linkage analyses. Am. J. Hum. Genet. 81, 559–575. 10.1086/519795 17701901 PMC1950838

[B88] Qi Z. Huang L. Zhu R. Xin D. Liu C. Han X. (2014). A high-density genetic map for soybean based on specific length amplified fragment sequencing. PLoS One 9, e104871. 10.1371/journal.pone.0104871 25118194 PMC4130620

[B89] Qiao R. Gao J. Zhang Z. Li L. Xie X. Fan Y. (2015). Genome-wide association analyses reveal significant loci and strong candidate genes for growth and fatness traits in two pig populations. Genet. Sel. Evol. 47, 17. 10.1186/s12711-015-0089-5 25885760 PMC4358731

[B90] Qin M. Li C. Li Z. Chen W. Zeng Y. (2020). Genetic diversities and differentially selected regions between Shandong indigenous pig breeds and Western pig breeds. Front. Genet. 10, 1351. 10.3389/fgene.2019.01351 32038711 PMC6987402

[B91] Quazi F. Molday R. S. (2013). Differential phospholipid substrates and directional transport by ATP-binding cassette proteins ABCA1, ABCA7, and ABCA4 and disease-causing mutants. J. Biol. Chem. 288, 34414–34426. 10.1074/jbc.M113.508812 24097981 PMC3843056

[B92] Rohrer G. A. Thallman R. M. Shackelford S. Wheeler T. Koohmaraie M. (2005). A genome scan for loci affecting pork quality in a Duroc-Landrace F population. Anim. Genet. 37, 17–27. 10.1111/j.1365-2052.2005.01368.x 16441291

[B93] Rubtsov A. M. Lopina O. D. (2000). Ankyrins. FEBS Lett. 482, 1–5. 10.1016/s0014-5793(00)01924-4 11018513

[B94] Škrlep M. Kavar T. Čandek-Potokar M. (2010). Comparison of PRKAG3 and RYR1 gene effect on carcass traits and meat quality in Slovenian commercial pigs. Czech J. Anim. Sci. 55, 149–159. 10.17221/6/2009-cjas

[B95] Su Y. H. Xiong Y. Z. Jiang S. W. Zhang Q. Lei M. G. Zheng R. (2004). [Mapping quantitative trait loci for meat quality trait in a Large White × Meishan cross]. Acta Genet. Sin. 31, 132–136. 15473302

[B96] Sun X. Liu D. Zhang X. Li W. Liu H. Hong W. (2013). SLAF-Seq: An efficient method of large-scale de novo SNP discovery and genotyping using high-throughput sequencing. PLoS One 8, e58700. 10.1371/journal.pone.0058700 23527008 PMC3602454

[B97] Thomsen H. Lee H. K. Rothschild M. F. Malek M. Dekkers J. C. M. (2004). Characterization of quantitative trait loci for growth and meat quality in a cross between commercial breeds of swine. J. Anim. Sci. 82, 2213–2228. 10.2527/2004.8282213x 15318717

[B98] Turner S. D. (2014). qqman: an R package for visualizing GWAS results using Q-Q and manhattan plots. Biorxiv 7, e1002043. 10.1101/005165

[B99] Van Deveire K. N. Scranton S. K. Kostek M. A. Angelopoulos T. J. Clarkson P. M. Gordon P. M. (2012). Variants of the ankyrin repeat domain 6 gene (ANKRD6) and muscle and physical activity phenotypes among European-derived American adults. J. Strength Cond. Res. 26, 1740–1748. 10.1519/JSC.0b013e31825c2bef 22580979 PMC4147939

[B100] Wang H. Wang X. Li M. Sun H. Chen Q. Yan D. (2022b). Genome-wide association study of growth traits in a four-way crossbred pig population. Genes (Basel). 13, 1990. 10.3390/genes13111990 36360227 PMC9689869

[B101] Wang H. Wang X. Yan D. Sun H. Chen Q. Li M. (2022a). Genome-wide association study identifying genetic variants associated with carcass backfat thickness, lean percentage and fat percentage in a four-way crossbred pig population using SLAF-Seq technology. BMC Genom 23, 594. 10.1186/s12864-022-08827-8 PMC9380336 35971078

[B102] Wang L. Zhang L. Yan H. Liu X. Li N. Liang J. (2014). Genome-wide association studies identify the loci for 5 exterior traits in a Large White × Minzhu pig population. PLoS One 9, e103766. 10.1371/journal.pone.0103766 25090094 PMC4121205

[B103] Wang W. H. Wang J. Y. Zhang T. Wang Y. Zhang Y. Han K. (2019). Genome-wide association study of growth traits in Jinghai Yellow chicken hens using SLAF-seq technology. Anim. Genet. 50, 175–176. 10.1111/age.12346 26365057

[B104] Wang W. Zhang T. Zhang G. Wang J. Han K. Wang Y. (2015). Genome-wide association study of antibody level response to NDV and IBV in Jinghai yellow chicken based on SLAF-seq technology. J. Appl. Genet. 56, 365–373. 10.1007/s13353-014-0269-y 25588649

[B105] Wimmers K. Murani E. Te Pas M. F. Chang K. C. Davoli R. Merks J. W. (2007). Associations of functional candidate genes derived from gene-expression profiles of prenatal porcine muscle tissue with meat quality and muscle deposition. Anim. Genet. 38, 474–484. 10.1111/j.1365-2052.2007.01639.x 17697135

[B106] Wu P. Wang K. Zhou J. Chen D. Yang X. Jiang A. (2020). Whole-genome sequencing association analysis reveals the genetic architecture of meat quality traits in Chinese Qingyu pigs. Genome 63, 503–515. 10.1139/gen-2019-0227 32615048

[B107] Xi Y. Xu Q. Huang Q. Ma S. Wang Y. Han C. (2021). Genome-wide association analysis reveals that EDNRB2 causes a dose-dependent loss of pigmentation in ducks. BMC Genom 22, 381. 10.1186/s12864-021-07719-7 PMC8146663 34034661

[B108] Xie D. Dai Z. Yang Z. Sun J. Zhao D. Yang X. (2017). Genome-wide association study identifying candidate genes influencing important agronomic traits of Flax (Linum usitatissimum L.) using SLAF-seq. Front. Plant Sci. 8, 2232. 10.3389/fpls.2017.02232 29375606 PMC5767239

[B109] Xie D. Dai Z. Yang Z. Tang Q. Sun J. Yang X. (2018). Genomic variations and association study of agronomic traits in flax. BMC Genom 19, 512. 10.1186/s12864-018-4899-z PMC6029072 29969983

[B110] Xiong X. Liu X. Zhou L. Yang J. Yang B. Ma H. (2015). Genome-wide association analysis reveals genetic loci and candidate genes for meat quality traits in Chinese Laiwu pigs. Mamm. Genome 26, 181–190. 10.1007/s00335-015-9558-y 25678226

[B111] Yang J. Lee S. H. Goddard M. E. Visscher P. M. (2011). Gcta: A tool for genome-wide complex trait analysis. Am. J. Hum. Genet. 88, 76–82. 10.1016/j.ajhg.2010.11.011 21167468 PMC3014363

[B112] Yang X. Deng F. Wu Z. Chen S. Y. Shi Y. Jia X. (2020). A genome-wide association study identifying genetic variants associated with growth, carcass and meat quality traits in rabbits. Anim. (Basel) 10, 1068. 10.3390/ani10061068 PMC7341332 32575740

[B113] Yu D. B. He Z. L. Zhang W. F. Jia X. X. Qiu X. S. Wang L. Y. (2008). The genetic effects of IGF2 gene intron3 variance in pigs. Yi Chuan 30, 87–93. 10.3724/sp.j.1005.2008.00087 18244908

[B114] Zhang L. C. Li N. Liu X. Liang J. Yan H. Zhao K. B. (2014). A genome-wide association study of limb bone length using a Large White × Minzhu intercross population. Genet. Sel. Evol. 46, 56. 10.1186/s12711-014-0056-6 25366846 PMC4219012

[B115] Zhao X. Wang C. Wang Y. Lin H. Wang H. Hu H. (2019). Comparative gene expression profiling of muscle reveals potential candidate genes affecting drip loss in pork. BMC Genet. 20, 89. 10.1186/s12863-019-0794-0 31791257 PMC6889219

[B116] Zhou X. Stephens M. (2012). Genome-wide efficient mixed-model analysis for association studies. Nat. Genet. 44, 821–824. 10.1038/ng.2310 22706312 PMC3386377

