# Supplementary figures and images for: Genome-wide association study reveals genetic loci and candidate genes for meat quality traits in a four-way crossbred pig population
Source: Front Genet. 2023 Feb 6;14:1001352. doi: 10.3389/fgene.2023.1001352 (PMC9939654; doi:10.3389/fgene.2023.1001352)

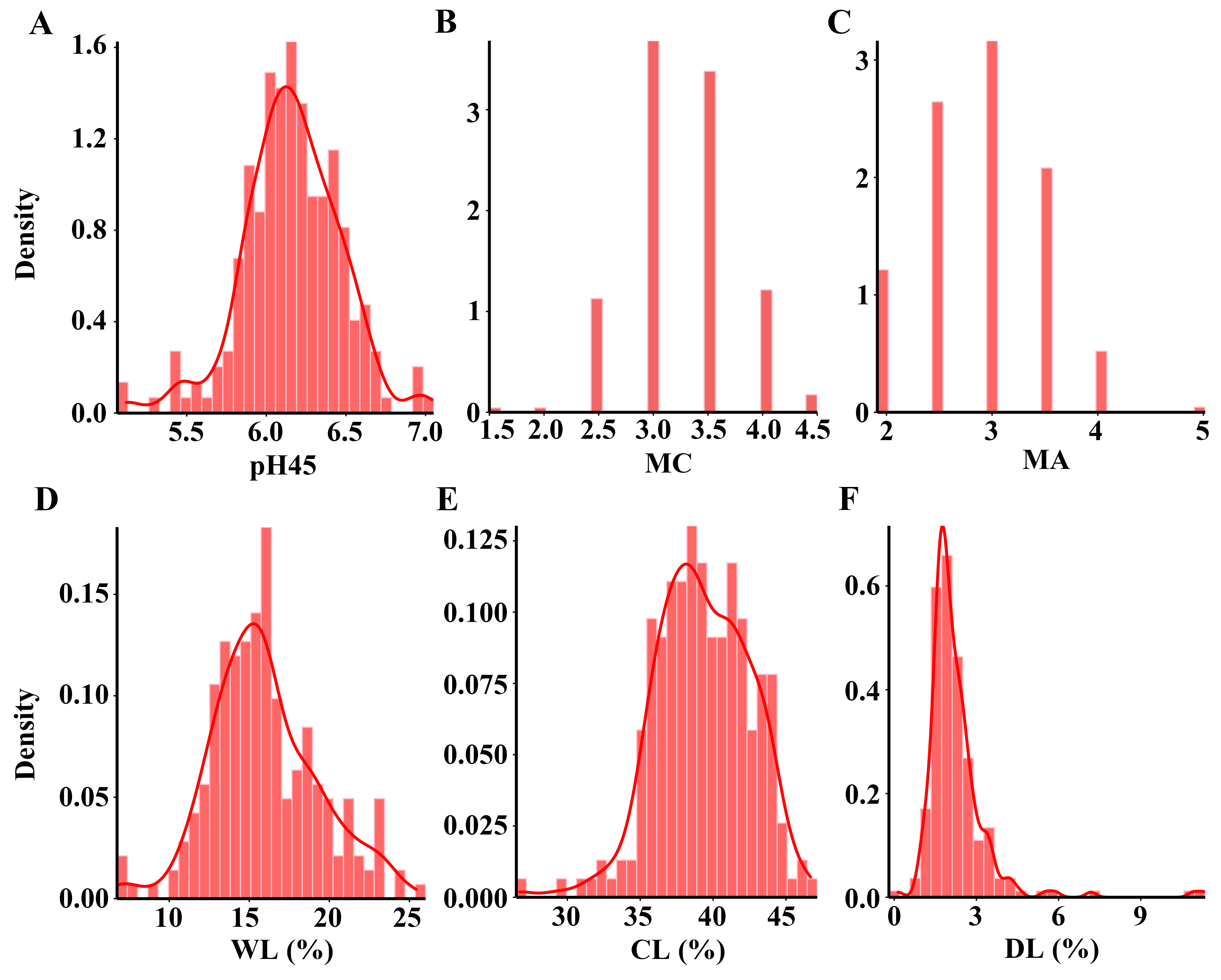

Supplement: Supplementary file 4 [file Image2.TIF]

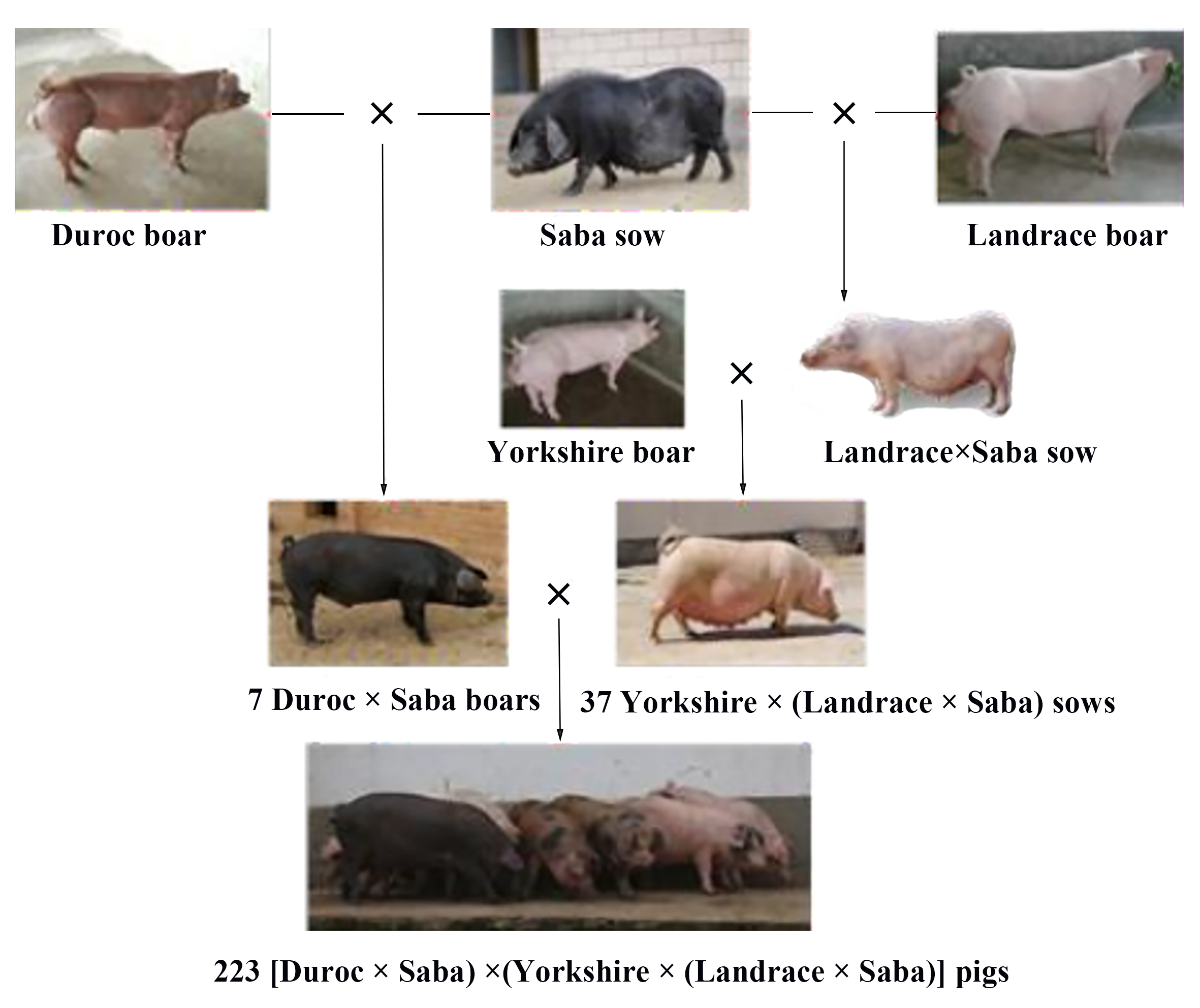

Supplement: Supplementary file 5 [file Image1.TIF]
